# An isoflavone catabolism gene cluster underlying interkingdom interactions in the soybean rhizosphere

**DOI:** 10.1093/ismeco/ycae052

**Published:** 2024-04-09

**Authors:** Noritaka Aoki, Tomohisa Shimasaki, Wataru Yazaki, Tomoaki Sato, Masaru Nakayasu, Akinori Ando, Shigenobu Kishino, Jun Ogawa, Sachiko Masuda, Arisa Shibata, Ken Shirasu, Kazufumi Yazaki, Akifumi Sugiyama

**Affiliations:** Research Institute for Sustainable Humanosphere, Kyoto University, Uji, Kyoto 611-0011, Japan; Research Institute for Sustainable Humanosphere, Kyoto University, Uji, Kyoto 611-0011, Japan; Faculty of Science, Hokkaido University, Sapporo, Hokkaido 060-0810, Japan; Research Institute for Sustainable Humanosphere, Kyoto University, Uji, Kyoto 611-0011, Japan; Research Institute for Sustainable Humanosphere, Kyoto University, Uji, Kyoto 611-0011, Japan; Research Institute for Sustainable Humanosphere, Kyoto University, Uji, Kyoto 611-0011, Japan; Division of Applied Life Sciences, Graduate School of Agriculture, Kyoto University, Kitashirakawa-oiwakecho, Sakyo-ku, Kyoto 606-8502, Japan; Division of Applied Life Sciences, Graduate School of Agriculture, Kyoto University, Kitashirakawa-oiwakecho, Sakyo-ku, Kyoto 606-8502, Japan; Division of Applied Life Sciences, Graduate School of Agriculture, Kyoto University, Kitashirakawa-oiwakecho, Sakyo-ku, Kyoto 606-8502, Japan; Plant Immunity Research Group, RIKEN Center for Sustainable Resource Science, Yokohama, Kanagawa 230-0045, Japan; Plant Immunity Research Group, RIKEN Center for Sustainable Resource Science, Yokohama, Kanagawa 230-0045, Japan; Plant Immunity Research Group, RIKEN Center for Sustainable Resource Science, Yokohama, Kanagawa 230-0045, Japan; Research Institute for Sustainable Humanosphere, Kyoto University, Uji, Kyoto 611-0011, Japan; Research Institute for Sustainable Humanosphere, Kyoto University, Uji, Kyoto 611-0011, Japan

**Keywords:** isoflavone, microbiota, catabolism, legume rhizosphere

## Abstract

Plant roots secrete various metabolites, including plant specialized metabolites, into the rhizosphere, and shape the rhizosphere microbiome, which is crucial for the plant health and growth. Isoflavones are major plant specialized metabolites found in legume plants, and are involved in interactions with soil microorganisms as initiation signals in rhizobial symbiosis and as modulators of the legume root microbiota. However, it remains largely unknown the molecular basis underlying the isoflavone-mediated interkingdom interactions in the legume rhizosphere. Here, we isolated *Variovorax* sp. strain V35, a member of the *Comamonadaceae* that harbors isoflavone-degrading activity, from soybean roots and discovered a gene cluster responsible for isoflavone degradation named *ifc*. The characterization of *ifc* mutants and heterologously expressed Ifc enzymes revealed that isoflavones undergo oxidative catabolism, which is different from the reductive metabolic pathways observed in gut microbiota. We further demonstrated that the *ifc* genes are frequently found in bacterial strains isolated from legume plants, including mutualistic rhizobia, and contribute to the detoxification of the antibacterial activity of isoflavones. Taken together, our findings reveal an isoflavone catabolism gene cluster in the soybean root microbiota**,** providing molecular insights into isoflavone-mediated legume–microbiota interactions.

## Introduction

Plants produce a vast array of low molecular weight organic compounds, known as plant specialized metabolites (PSMs). These metabolites are not directly involved in plant development and reproduction but largely contribute to plant–environment interactions and adaptation [1, 2]. Over the past decade, it has been demonstrated that PSMs are crucial for the interactions between plants and their associated microbial communities, also known as plant microbiota [3–6]. Notably, the bacterial metabolic capacity of PSMs plays a key role in PSM-mediated host–microbiota interactions. Root microbiota members have the ability to degrade and/or utilize host–specific PSMs, conferring competitive advantages on their root colonization and contributing to the assemblage of species-specific root microbiota [7–9]. Therefore, discovering the bacterial catabolic pathways of PSMs is crucial for elucidating the molecular mechanisms of plant–microbe interactions.

Isoflavones are a subgroup of flavonoids that are mainly found in *Fabaceae* [10]. They play a crucial role in establishing nodule symbiosis, and assembling root microbiota [11]. Additionally, they serve as phytoalexins by suppressing the growth or spore germination of pathogens [12, 13]. Isoflavone synthase, which belongs to the cytochrome P450 family, is a key enzyme for isoflavone biosynthesis. It catalyzes the hydroxylation at C-2 accompanied by the 1,2-aryl migration from C-2 to C-3 [14]. Subsequent enzymatic reactions lead to the formation of various isoflavone derivatives [15–18]. Daidzein, a major isoflavone in soybean (*Glycine max*), is secreted into the rhizosphere in response to nitrogen deficiency [19]. This secretion induces the expression of *nod* genes of the mutualistic rhizobia, *Bradyrhizobium japonicum* [20, 21], and modulates the soybean root microbiota [22, 23].

In the human gut, daidzein is metabolized by intestinal bacteria and converted to equol, which exhibits great estrogenic and antioxidant activity [24, 25] or *O*-desmethylangolensin (*O*-DMA) [26]. In this pathway, daidzein is metabolized to dihydrodaidzein by a racemase and a reductase. The resulting dihydrodaidzein is further metabolized to equol by a series of reductase reactions or converted to *O*-DMA via the fission of the heterocyclic C-ring. Genes involved in human gut daidzein catabolism have been identified from several intestinal bacteria, including *Bifidobacterium pseudolongum*, *Clostridium* sp., and *Slackia isoflavoniconvertens* [27–29]. It is believed that differences in the composition of these bacterial species are responsible for the metabolic variation of intestinal daidzein [30].

Certain rhizobia species have been reported to degrade isoflavones in soil microorganisms through C-ring calving, producing degradation intermediates such as resorcinol, *p*-coumaric acid, and *p*-hydroxybenzoic acid [31]. However, it is important to note that this catabolism pathway is distinct from that of human gut microorganisms, and no genes involved in isoflavone catabolism have been identified in soil microorganisms. We previously demonstrated that microorganisms in the soybean rhizosphere degrade daidzein [32]. The addition of daidzein to soil led to the enrichment of *Comamonadaceae*, which is a predominant bacterial family in soybean roots [23]. *Comamonadaceae* harbor genomic features that enable the catabolism of an array of aromatic compounds [33], implying that these bacterial species are potentially responsible for isoflavone degradation in soybean roots.

In this study, we isolated isoflavone-degrading *Variovorax* spp., soil bacteria belonging to Comamonadaceae, from soybean roots and identified the gene cluster responsible for isoflavone catabolism, which we named “the isoflavone catabolism” (*ifc*) gene cluster. By integrating comparative genome analysis and an *in vitro* fitness assay, we demonstrated that the *ifc* gene cluster underlies the interactions between legumes and their root microbiota.

## Materials and methods

### Bacterial strains and chemicals

The bacterial strains and plasmids used in this study are listed in Dataset S2. The bacterial strains belonging to *Comamonadaceae* were isolated from soybean roots grown in nitrogen-deficient conditions. A detailed description and isolation procedures can be found in Yazaki *et al*. [34]. All *Variovorax* isolates were grown at 28°C in peptone-beef extract medium (10 g/l peptone, 10 g/l beef extract, and 5 g/l NaCl) supplemented with appropriate antibiotics (5 μg/ml tetracycline). *Escherichia coli* strains were grown at 37°C in Luria–Bertani medium supplemented with appropriate antibiotics (50 μg/ml kanamycin and 5 μg/ml tetracycline). Chemicals were obtained from Wako Pure Chemical Industries (Japan) or Nacalai Tesque (Japan), otherwise listed in Dataset S2.

### Plasmid construction and generation of gene knockout mutants

All constructions and primers used in this study are listed in Dataset S2. All insertion and deletion mutants were generated by homologous recombination. The constructed vectors were introduced into strain V35 by triparental mating*.* Detailed methods can be found in Supplemental Methods.

### Measurements of isoflavone-degrading activities using resting cells

Bacterial strains were cultured overnight at 28°C in 2 ml of peptone-beef extract medium. Bacterial cells were collected by centrifugation at 1500 x *g* for 10 min and washed twice with mineral salt (MS) medium [35]. Bacterial pellets were resuspended in 2 ml of MS medium supplemented with daidzein at a concentration of 20 μM and incubated for 3 days. After centrifugation at 1500 x *g* for 10 min, supernatants were collected and stored at −80°C until use. To extract isoflavones from the supernatants, 500 μl of supernatant was acidified with 5 μl of 1 M HCl and mixed with 500 μl of ethyl acetate. After centrifugation at 9100 x *g* for 10 min, 400 μl of organic layer was collected and evaporated. The extracts were resuspended in 95% MeOH containing 0.1% (v/v) formic acid. After filtering through a 0.45-μm Minisart RC4 filter (Sartorius, Germany), collected samples were stored at −30°C until ultra-high-performance liquid chromatography (UPLC) analysis. Detailed methods for UPLC analysis can be found in Supplemental Methods.

### Time course daidzein degradation activity of strain V35

Bacterial strains were cultured overnight at 28°C in 2 ml of peptone-beef extract medium. Bacterial cells adjusted to OD_600_ = 1.0 were collected by centrifugation at 13000 x *g* for 1 min and washed twice with MS medium. The resulting bacterial pellet was resuspended in 2 ml of MS medium supplemented with daidzein a concentration of 20 μM and cultured at 28°C. For the time course daidzein degradation assay of the V35 wild-type strain, 100 μl of culture supernatant was collected at 0, 1, 2, 4, and 8 h, immediately mixed with equivalent volume of methanol, and centrifuged at 13000 x *g* for 1 min. Daidzein degradation of *ifc* mutants was conducted for 48 h of cultivation. After filtration through a 0.45-μm Minisart RC4 filter (Sartorius, Germany), collected samples were stored at −30°C until UPLC and LC–MS analysis. Detailed methods for UPLC and LC–MS analysis can be found in Supplemental Methods.

### Heterologous expression and measurement of enzymatic activity of recombinant proteins in *E. coli*

The enzymatic activity of Ifc enzymes was characterized using heterologous expression system using *E. coli* strain BL21 (DE3). Expressed enzymes were purified by Ni-NTA chromatography and subjected to *in vitro* assay. Detailed methods for heterologous expression of recombinant proteins and enzymatic reaction measurements can be found in Supplemental Methods.

### RNA extraction and RNA-seq analysis

RNA-seq analysis was conducted with strain V35 cultured in MS medium or MS medium containing daidzein at final concentration of 30 μg/ml and cultured for 16 h at 28°C. Total RNA was extracted using TRI Reagent (Cosmo Bio Co., Ltd, Japan) according to the manufacturer’s instructions and purified by phenol–chloroform extraction. The extracted RNA was sent to Macrogen (Korea) for library preparation and sequencing using the NovaSeq 6000 (Illumina, USA). Obtained raw reads were mapped to strain V35 genome using Kallist 0.43.1 with the default setting [36]. The raw sequence reads were submitted to the DNA Data Bank of Japan (PRJDB16019) to be publicly available. Detailed methods for RNA extraction and sequencing analysis can be found in Supplemental Methods.

### Whole genome sequencing and comparative genome analysis

The genomic DNA extraction, whole-genome sequencing, and subsequent assembly were performed as described previously [8]. Comparative genome analysis was conducted by integrating the public genome sequences of *Variovorax* retrieved from the Integrated Microbial Genomes & Microbiomes system (https://img.jgi.doe.gov) and the National Center for Biotechnology Information (NCBI) (https://www.ncbi.nlm.nih.gov). A full description of methods and algorithms used in this study can be found in Supplemental Methods.

### *In vitro* bacterial growth assay

V35 and V35 Δ*ifcA* were cultured in 3 ml of peptone-beef extract medium for 16 h at 28°C with shaking at 180 rpm. The overnight cultures were diluted in peptone-beef extract medium and adjusted to OD_600_ = 0.001. Different concentrations of daidzein (10, 30, 100 μM) were added to the bacterial cultures and applied into a 96-well microplate. Growth curves were monitored under the wavelength of OD_600_ nm with an interval of 30 min at 28°C, by Synergy HTX (BioTek Instruments, USA). For statistical analysis, we calculated area under the curve for each sample and normalized it by that of control (0 μM of daidzein). For the growth assays with daidzein as the sole carbon source, the overnight cultures were washed and diluted in minimal media consisted of 3 g/l (NH_4_)_2_HPO_4_, 1 g/l KCl, and 0.1 g/l MgSO4・7H_2_O, and adjusted to OD_600_ = 0.001. The minimal media was supplemented with daidzein or glucose to reach a final concentration of 1200 μM for daidzein and 750 μM for glucose.

## Results

### Isolation of soybean root-associated bacteria involved in isoflavone catabolism

**Figure 1:**
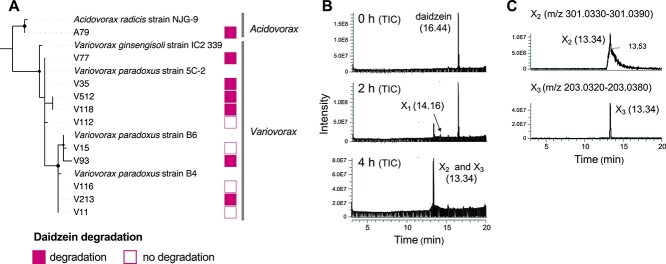
Phylogenetic analysis of soybean root-associated bacteria belonging to family *Comamonadaceae* and daidzein catabolism intermediates in *Variovorax* sp. strain V35; (A) the phylogenic tree was constructed using the maximum likelihood estimation (MLE) method using 10F/800R region of 16S rRNA gene sequences; bootstrap values (1000 replicates) above 0.8 are shown in nodes; the daidzein degradation ability of each isolate is represented by filled and empty squares, respectively; (B) LC–MS analysis of the culture supernatant at 0, 2, and 4 h from the V35 culture supernatant grown in daidzein supplemented MS medium; total ion chromatograms obtained in negative ionization mode with full-scan range of *m/z* 50–350 are shown.

We previously reported enrichment of the family *Comamonadaceae* in soil treated with daidzein [23] and isolated strains of two *Comamonadaceae* bacterial genera, *Variovorax* and *Acidovorax*, from soybean roots (Fig. 1A) [34]. Among these isolates, seven strains degraded daidzein (Fig. 1A and Supplementary Fig. S1). Phylogenetic analysis based on the 16S rRNA gene sequence revealed that *Variovorax* isolates were closely related to *Variovorax paradoxus* (Fig. 1A)*.* Daidzein-degrading strains were found regardless of their phylogenic distance. Due to its high mutagenesis efficiency, we selected strain V35 for further analysis.

To identify the daidzein degradation intermediates, we analyzed time-course daidzein degradation of strain V35 by high-resolution mass spectrometer (Fig. 1B). This strain completely degraded daidzein within 4 h after inoculation and produced three reaction product candidates (compounds X_1_–X_3_, Fig. 1B). Total ion current chromatogram analysis in the range of mass-to-charge ratio (*m/z*) of 50–350 detected two peaks in the supernatant at the retention times (Rt) of 13.34 and 14.16. The compound at Rt 14.16 (compound X_1_) showed an ion at *m/z* 269.0455 (Supplementary Fig. S2A), consistent with the calculated mass of C_15_H_10_O_5_ (269.0450). This suggests that this compound may be the first reaction product in the daidzein catabolism pathway of this strain, formed by mono-hydroxylation of daidzein. The peak observed at Rt 13.34 consisted of a sharp peak and a broad peak (compounds X_2_ and X_3_, Fig. 1C), with mass fragment ions at *m/z* 203.0349 and 301.0352, respectively (Supplementary Fig. S2B and C). Compound X_3_ with an *m/z* of 203.0349 had a lower molecular weight than the other two intermediates, suggesting that this compound is a late intermediate. These results suggest that the family *Comamonadaceae*, particularly the genera *Variovorax* and *Acidovorax*, are responsible for the isoflavone catabolism in the soybean rhizosphere.

### Identification of the isoflavone catabolism gene cluster

**Figure 2:**
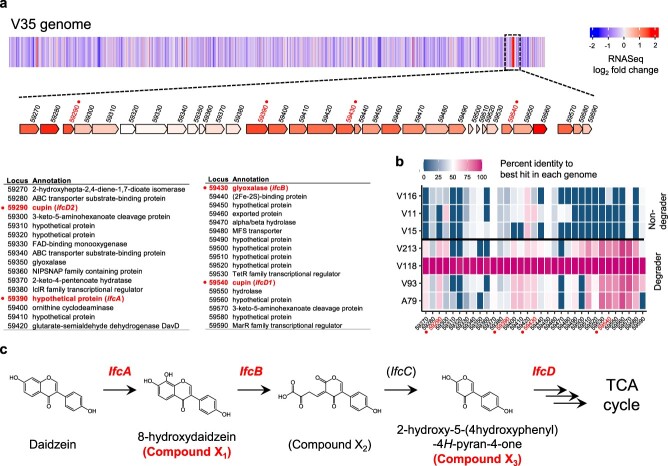
Screening of isoflavone catabolism gene cluster and proposed isoflavone catabolism pathway of genus *Variovorax*; (A) a heatmap of the RNA-seq experiment was denoted on the linear chromosome map of strain V35, colored by the log_2_-transformed-fold change in gene expression during the growth in presence of daidzein versus absence of daidzein; the detected hotspot region is displayed on the gene map colored by the log_2_-transformed-fold change; characterized *ifc* genes are indicated with dots; the functional assignments annotated are shown in the table below the gene map; (B) percent identity of each *ifc* gene to the genes from *Variovorax* isolates is shown in heat map; characterized *ifc* genes are indicated with dots; (C) isoflavones were oxidatively catabolized by *ifc* genes and eventually entered the citrate cycle; the *ifc* genes and isoflavone catabolism intermediates identified in this study are represented.

To identify isoflavone catabolism genes, we sequenced the genomes of *Variovorax* and *Acidovorax* isolates. We obtained 10 complete genome sequences, ranging from one to three closed contigs per strain, and one near-complete genome (Dataset S1A). Pairwise average nucleotide identity analysis showed that strain V118 had almost identical genome sequences with V512 and V77 (Supplementary Fig. S3). We removed strains V512 and V77 from further analysis. Strain V35 degraded daidzein at a faster rate when they were precultured in daidzein-containing medium than in control medium (Supplementary Fig. S4), indicating the induction of isoflavone catabolism genes by daidzein. Additionally, we conducted comparative transcriptome analysis of strain V35 grown in the presence and absence of daidzein. By integrating comparative transcriptome and genome analysis, we screened for the candidate genes that were (1) upregulated in the presence of daidzein, (2) highly conserved in the daidzein-degrading strains, V213, V118, V93, and A79, and (3) less conserved in the daidzein non-degrading strains, V116, V11, and V15. This analysis identified one hotspot (from gene locus 59270 to locus 59590) that was composed of catabolism-related genes such as oxidoreductases, hydrolases, transcriptional regulators, and transporters (Fig. 2A). All genes in the hotspot were upregulated in the presence of daidzein (Fig. 2A), and tend to be conserved among daidzein-degrading strains (Fig. 2B).

Within the hotspot, we found that loci 59290, 59390, 59430, 59470, and 59540 showed amino acid sequence similarities to flavonoid degradation (*fde*) genes from *Herbaspirillum seropedicae* SmR1 (Supplementary Fig. S5). These genes are responsible for the early stage of naringenin degradation [37, 38]. Locus 59390 and locus 59430 were predicted to encode a putative flavin adenine dinucleotide (FAD)-binding monooxygenase and a putative glyoxalase, respectively. These proteins shared amino acid sequence similarities to *fdeE* (36.3% identity with 95% coverage) and *fdeC* (65.8% identity with 99% coverage), respectively, both of which are involved in the hydroxylation and dioxygenation of the A-ring of naringenin. Locus 59470 encodes alpha/beta hydrolase and showed sequence similarity with 2,6-dihydropseudooxynicotine hydrolase (25% identity with 84% coverage) from *Arthrobacter nicotinovorans* (Q93NG6), which cleaves a C–C bond in 2,6-dihydroxypseudooxynicotine [39]. Both locus 59540 and locus 59290 encode proteins belonging to the cupin superfamily, which is characterized by a conserved barrel domain and consists of functionally diverse proteins such as isomerases, oxidoreductases, and seed storage proteins [40]. In addition, locus 59540 and locus 59290 shared 65% sequence identity, suggesting that these enzymes are homologous proteins. We hypothesized that this hotspot is a putative gene cluster for isoflavone catabolism and designated it as the *isoflavone catabolism* (*ifc*) gene cluster.

### Identification of isoflavone catabolism genes via mutagenesis study

**Figure 3:**
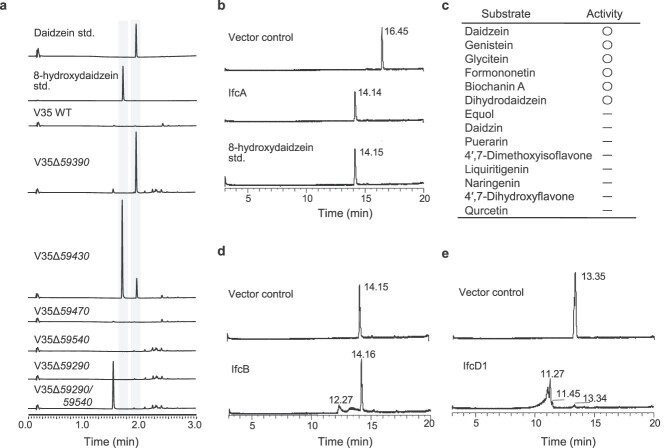
Daidzein degradation assay of *ifc* mutants and enzymatic activity of Ifc enzymes; (A) UPLC analysis of culture supernatant of each *ifc* mutant grown in daidzein-supplemented MS medium; essentially identical results were obtained in two independent experiments; (B, D, and E) LC–MS analysis of the reaction products from the recombinant proteins of IfcA, IfcB, and IfcD; a crude protein from *E. coli* transformed with an empty pET22b or pCold ProS2 vector was used as the negative control; total ion current chromatogram obtained in negative ionization mode with a full-scan range of *m/z* 50–350 is shown; essentially identical results were obtained in two independent experiments; (C) substrate specificity of IfcA; the catalytic activities of IfcA for each substrate are shown; data were obtained from three technical replicates.

To validate the involvement of the candidates in daidzein catabolism, we generated gene-disruption mutants of genes in the *ifc* gene cluster listed in Dataset S2. We found that, unlike the wild-type, V35 Δ*59390* was not able to degrade daidzein, indicating that locus 59390 encodes the initiating enzyme of the isoflavone catabolism pathway (Fig. 3A). In contrast, V35 Δ*59430* and the double mutant V35 Δ*59290/*Δ*59540* partially degraded daidzein and produced distinctive peaks at Rt 1.7 and 1.5 min, respectively, compared to the wild-type (Fig. 3A), indicating that these genes are also involved in daidzein catabolism. Notably, single mutants of locus 59290 and locus 59540 retained the ability to degrade daidzein, suggesting that locus 59540 and locus 59290 are functionally redundant. V35 Δ*59430* accumulated a compound which was consistent with the compound X_1_ (Fig. 2A and Supplementary Fig. S6). The Rt and mass spectrum of the compound X_1_ were identical to the authentic standard of 8-hydroxydaidzein (Supplementary Fig. S7). We also found that the double mutant of locus 59540 and locus 59 290 accumulated a distinctive peak at Rt 13.35 min, with a mass fragment ion at *m/z* 203.0353 (Supplementary Fig. S8). The retention time and mass spectrum of the peak accumulated in V35 Δ*59290/*Δ*59540* corresponded to the compound X_3_. Among the *ifc* catabolism intermediates, the compound X_3_ stably accumulated in the supernatant and remained after 48 h of cultivation. Using nuclear magnetic resonance (NMR) analysis, we determined the chemical structure of compound X_3_ as 2-hydroxy-5-(4-hydroxyphenyl)-4*H*-pyran-4-one, a novel isoflavone catabolite (Supplementary Fig. S9). Gene disruption of locus 59470 did not affect the daidzein degradation ability. Collectively, even though the gene associated with the conversion of compound X_2_ to X_3_ was remained to be identified, we demonstrated that the genes located on locus 59390, 59430, 59540, and 59290 are isoflavone catabolism genes. We have named these genes *ifcA*, *B*, *D1*, and *D2*, respectively.

### Heterologous expression and functional characterization of the Ifc enzymes

We further characterized the catalytic activities of the recombinant Ifc enzymes expressed in *E. coli*. When purified IfcA was incubated with daidzein in the presence of nicotinamide adenine dinucleotide phosphate (NADPH), a reaction product was detected. This product was identified as 8-hydroxydaidzein by direct comparison with the standard specimen (Fig. 3B and Supplementary Fig. S10), indicating that IfcA catalyzes hydroxylation of the 8-position of daidzein. This reaction was dependent on the presence of NADPH (Supplementary Fig. S11). We also examined the substrate specificity of IfcA using representative isoflavone aglycons, isoflavone glycosides, and other flavonoid families. IfcA reacted with a series of isoflavones aglycons, including genistein, glycitein, formononetin, biochanin A, and dihydrodaidzein. However, IfcA did not catalyzed 4′,7-dimethoxyisoflavone, equol, isoflavone glycoside, or any other flavones, flavanones, or flavonols (Fig. 3C and Supplementary Fig. S12). This result suggests that IfcA is specific to isoflavone aglycons and that the methoxy moiety at the 7-position and sugar moiety at the 7- and 8-position may inhibit substrate recognition and/or the catalytic activity of this enzyme. We also analyzed whether wild-type V35 and the Δ*ifcA* mutant degrade isoflavones secreted from soybean roots. The wild-type strain V35 degraded genistein in addition to daidzein, but the Δ*ifcA* mutant did not degrade these isoflavones (Supplementary Fig. S13A and B). Although recombinant IfcA accepted glycitein as a substrate, both wild-type V35 and Δ*ifcA* mutant did not degrade glycitein (Supplementary Fig. S13C), suggesting a different property of glycitein compared to daidzein in its transport into the cells and/or activation of the *ifc* gene cluster. IfcA belongs to group A flavin-containing monooxygenases (FMOs), a flavoprotein family that binds the FAD cofactor and NAD(P)H coenzyme [41]. However, this enzyme forms a distinctive clade (supported by an 84.5% bootstrap portion: Supplementary Fig. S14), indicating that IfcA represents a distinctive class of the group A FMOs that specifically catalyzes isoflavones.

Although a peak with a mass spectrum identical to the compound X_2_ was observed following the enzymatic reaction of recombinant IfcB protein with 8-hydroxydaidzein as a putative substrate (Supplementary Fig. S15), 8-hydroxydaidzein remained after the reaction (Fig. 3D).

When IfcD1 was incubated with the compound X_3_ as a substrate, two peaks were detected with Rts of 11.27 and 11.45 min, respectively, and mass fragment ions were given at *m/z* 177.0555 and 193.0505, respectively (Fig. 3E and Supplementary Fig. S16). Similarly, the reaction of IfcD2 with the compound X_3_ also gave the same two products (Supplementary Fig. S17), further confirming the functional redundancy between IfcD1 and IfcD2. To further characterize the enzymatic reactions of IfcD, as well as to estimate the chemical structure of the reaction products, we synthesized two stable isotopes of the compound X_3_, both of which are isotopically labeled at 3′ and 5′ or 2′, 3′, 5′, and 6′ positions, by biotransformation using the V35 Δ*ifcD1/D2* mutant. These compounds were named X_3_-d_2_ and X_3_-d_4_, respectively (Supplementary Figs S18A and 19A). When IfcD1 was incubated with compound X_3_-d_2_ and X_3_-d_4_, the products had *m/z* values that were two and four mass units higher than those reacted with non-labeled compound X_3_, suggesting that compound X_3_ retained the B–ring of daidzein after reaction with IfcD1 (Supplementary Figs S18B and C, and S19B and C). The exact mass of the resulting compounds corresponded with the calculated mass of C_10_H_10_O_3_ (177.0552) and C_10_H_10_O_4_ (193.0501), respectively. Thus, we predicted the chemical structures of these compounds (Supplementary Fig. S20). These results collectively illustrated a novel oxidative isoflavone catabolism pathway in the genus *Variovorax* (Fig. 2C).

### Distribution of the *ifc* genes in bacterial kingdom

**Figure 4:**
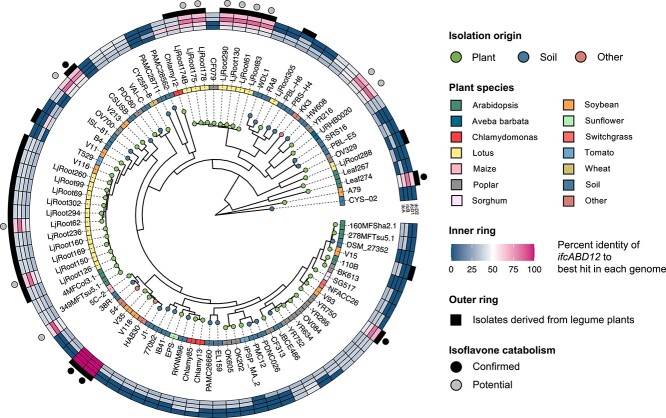
Phylogenetic distribution of the *ifc* genes in genus *Variovorax*; the phylogenetic tree of 88 *Variovorax* genomes was inferred from aligned single-copy genes using the MLE method; leaf nodes are colored by the isolation source of each genome; the inner ring depicts the plant species from which each strain isolated; heatmap representing percent identity of BLASTP hits in each *Variovorax* genome to IfcA/B/D1/D2 from strain V35; the black outer rings represent the strains isolated from legume plants; outer circles indicate the isoflavone-degrading strains; black color indicates the isoflavone degrading strains whose degradation abilities were experimentally confirmed; gray color indicates the potential isoflavone-degrading strains predicted by *in silico* analysis.

**Figure 5:**
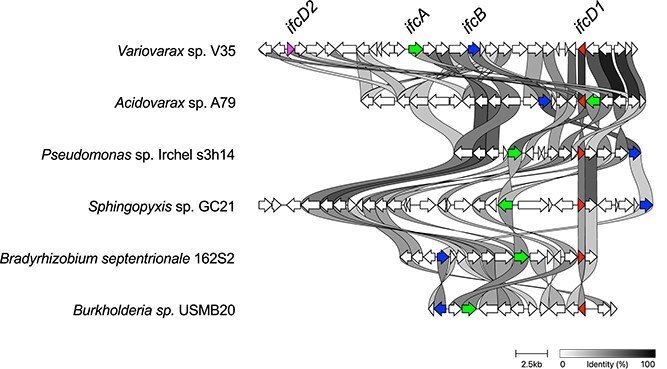
Synteny analysis of the *ifc* gene cluster between taxonomically distinct bacterial species; synteny analysis and visualization were performed using clinker [61]; percent identity of shared genes between the *ifc* genes of strain V35 and homologous genes from each bacteria strain is represented in greyscale.

To assess the function of the *ifc* genes in interactions with host legume plants, we established a *Variovorax* pan-genome by integrating eight of our isolates (*Acidovorax* sp. strain A79 was used as outgroup) and 80 publicly available genome sequences of *Variovorax* (Dataset S1B). We confirmed that the selected bacteria strains do not share identical genomic features by comparing their proteome profiles using OrthoFinder2. Most of the *Variovorax* strains were isolated from soil or plant environments. The *Ifc* genes were uniquely found in the strains isolated from plant-associated environments, especially from soybean and *Lotus japonicus* roots rich in isoflavones (*P* = 4.60 × 10^4^ by Fisher’s exact test) (Fig. 4). The *Ifc* genes were also identified from strains isolated from non-legume plants, such as maize, poplar, and *Avena barbata*. We further investigated the presence of *ifcA* in the bacterial kingdom by BLASTP search using IfcA as a query against the protein database at the NCBI. IfcA homologs were uniquely identified from soil bacteria belonging to phylum *Proteobacteria*, including *Variovorax*, *Burkholderia*, *Paraburkholderia*, *Bradyrhizobium*, *Pseudomonas*, and *Sphingopyxis*, but not found in intestinal bacteria species (Supplementary Fig. S21), and the *ifc* including *ifcA/B/D1/D2* was conserved in those strains (Fig. 5). Phylogenetic analysis of IfcA further demonstrated that IfcA was divided into two distinctive groups. Even though further functional characterization is needed, the two IfcA groups may have functional differences, such as substrate specificity.

**Figure 6:**
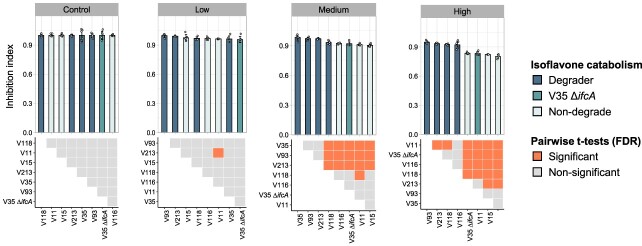
*In vitro* growth assay of *Variovorax* isolates and *ifcA*-deficient mutant; the upper barograph represents the mean of inhibition index ±SD (*n* = 6); control, 0 μM of daidzein; low, 10 μM of daidzein; middle, 30 μM of daidzein; high, 100 μM of daidzein; lower panel displays the corresponding statistical analysis of pairwise *t*-tests; significant (FDR-adjusted *P* values <.05) and non-significant (FDR adjusted *P* values >.05).

To test whether the *ifc* gene cluster is involved in the bacterial adaptation to the isoflavone containing environment, we measured the *in vitro* bacterial growth of all *Variovorax* isolates and an *ifcA*-deficient mutant in nutrient media with three different concentrations of daidzein. At high concentration (100 μM), daidzein significantly inhibited the growth of isoflavone-non-degrading strains, while no clear growth inhibition was observed in isoflavone-degrading strains (Fig. 6 and Supplementary Fig. S22). It is worth noting that the growth of the V35 Δ*ifcA* mutant was also suppressed to a comparable extent as that of non-degrading strains. Although the wild-type V35 strain was able to grow in the minimal medium containing daidzein as the sole carbon source, the Δ*ifcA* mutant was not able to grow in this medium (Supplementary Fig. S23). The difference in bacterial growth was not observed when they were grown in the minimal medium supplemented with glucose as the carbon source. Functional annotation using KofamKOALA showed that *Variovorax* isolates possessed gene sets involved in a meta-cleavage of catechol pathway (Supplementary Fig. S24). This pathway cleaves the aromatic ring of catechol and eventually enters the tricarboxylic acid cycle. These results demonstrate that isoflavone has a dual function in the legume rhizosphere, acting as both an antibacterial agent and carbon source. Collectively, our findings imply that the *ifc* gene cluster contributes to the bacterial adaptation to legume roots by facilitating the utilization and detoxification of isoflavones.

## Discussion

PSMs play pivotal roles in the interaction with plant microbiota as a signaling molecule initiating symbiotic interactions with mutualistic microorganisms and as a modulator for the community structure of root microbiota. However, molecular mechanisms underlying the metabolic interactions between plants and its associated microbiota remain largely unknown. Here, we isolated isoflavone-degrading soil bacteria from soybean roots and discovered a gene cluster for isoflavone oxidative catabolism, providing molecular insights into the PSMs-mediated interkingdom interactions.

Several flavonoid catabolism pathways have been identified from soil bacterial species. Quercetinases identified from *Streptomyces* sp. FLA, and *Bacillus subtilis* YxaG, for example, catalyze the 2,4-dioxygenolytic cleavage of quercetin [42, 43]. *Pseudomonas putida* PML2 catabolizes quercetin by C-ring cleavage starting from 3,3′-didehydroxylation [44]. Although flavonoid catabolism in these bacteria species is initiated by C-ring cleavage, *fde* genes in *H. seropedicae* SmR1 encode enzymes that catabolize naringenin by 8-hydroxylation and subsequent dioxygenation of the A-ring [38]. These findings illustrate that bacterial oxidative flavonoid catabolism is distinctively divided into either A- or C-ring cleaving pathways.

We found the A-ring cleaving isoflavone catabolism pathway of *Variovorax*. Although additional combinational mutagenesis studies are required to eliminate the possibility of pathway branching and/or alternative substrate inputs, this catabolic pathway resembles the naringenin degradation by the *fde* genes in *H. seropedicae* SmR1 [38]. In contrast, it has been reported that *Rhizobium* sp. strain NGR234 degraded daidzein via C-ring cleavage fission [31], implying another oxidative isoflavone catabolism pathway initiated from C-ring cleavage in soil bacteria.

Comparative genome analysis revealed that *Variovorax* strains isolated from the roots of legume plants such as soybean and *L. japonicus* often possess the *ifc* genes (Fig. 4). This observation is in line with our previous report that *Arthrobacter* isolates derived from tobacco roots harbor a characteristic genomic feature, enabling them to utilize tobacco-specific PSMs such as nicotine and santhopine [8]. Additionally, *Sphingobium* isolates derived from tomato roots have genes for utilizing α-tomatine, a predominant PSM in tomato rhizosphere [9]. Similarly, the metabolic combinations between PSMs and the corresponding catabolism capacity of the associated root microbiota have also been found in maize and sesame [45, 46]. These results indicate that the PSM catabolic ability of plant-associated microorganisms plays a crucial role in their interaction with host plants and in the formation of plant species-specific root microbiota. The bacterial catabolic abilities of PSMs may contribute to their predominant colonization in the roots and rhizosphere, where PSMs in general inhibit the growth of microorganisms [12, 47, 48]. Maize root microbiota showed a higher tolerance toward benzoxazinoids (BXs), which are antimicrobial compounds produced by maize, when compared to the bacterial strains isolated from *Arabidopsis* roots [49]. Additionally, some of the maize microbiota are capable of degrading BXs and possess a gene cluster responsible for BXs catabolism [45]. We demonstrated that stain V35 uses daidzein as a carbon source and that disruption of the *ifcA* gene reduced the growth of V35 strain in media containing daidzein (Fig. 6). The *ifc* gene cluster was also detected in strains derived from non-legume plants. Although isoflavones are typically associated with legumes, they are also produced by a wide range of non-legume plants, including both dicot and monocot [50]. This suggests that the *ifc* genes may be involved in the interaction with isoflavone-producing non-legume plants. Synthetic community approach designed with a phylogenetically diverse range of bacterial isolates and their mutants together with metagenomic analysis to reveal the distribution of the *ifc* gene cluster will provide direct evidence of the involvement of the* ifc* gene cluster in colonizing host roots. Future studies should also investigate genomic features beyond the *ifc* gene cluster, including those related to chemotaxis and root colonization.

Our comparative genome analysis revealed that the *ifc* genes are distributed in phylum *Proteobacteria*, including *Bradyrhizobium* and *Burkholderia* species, which are known to establish a symbiotic relationship with legume plants [51, 52]. Interestingly, *Burkholderia* strains possessing the *ifc* genes, such as *Burkholderia* sp. USMB20 and *Paraburkholderia steynii*, have been isolated from legume nodules [53, 54]. It has been reported that flavonoid metabolites produced by symbiotic rhizobia affect *nod* gene expression and bacterial growth. Chalcones, a luteolin degradation intermediate of *Rhizobium meliloti* [31], exhibits stronger induction activity of *nod* genes compared to luteolin [55]. *Bradyrhizobium* sp. strain ORS285 converted naringenin into its *O*-methylated form, which stimulates the growth of strain ORS285 [56]. In symbiotic interaction, the utilization and detoxification of metabolites present in the host root exudates are critical. A mutant of *R. meliloti*, which had impaired a proline metabolism, showed reduced nodulation efficiency and competitiveness in alfalfa roots [57]. *Mesorhizobium tianshanense* detoxifies canavanine, an antimicrobial compound produced by legume plants, by exporting this metabolite [58]. Together with our finding that the *ifc* gene cluster is involved in the bacterial growth in isoflavone-containing environments, these results suggest that the *ifc* gene cluster may confer an advantage to rhizobia for colonizing and surviving in host roots.

Plant microbiota play pivotal roles in plant growth and tolerance to various stresses. Designing synthetic microbiota in agriculture is highly appreciated to reduce the extreme dependence on fossil-fuel derived fertilizers and pesticides, while the molecular mechanisms underlying microbial competitiveness and colonization to roots remain largely unknown [59, 60]. Our findings set a new trajectory toward a molecular understanding of plant–microbiota interactions and their potential application in sustainable agriculture through the combination of microorganisms and metabolites that they metabolize.

## Supplementary Material

Dataset_S1_ycae052

Dataset_S2_ycae052

Supplemental_Materials_ISME_v2_ycae052

## Data Availability

Nucleotide sequences of *ifcA* (LC777269), *ifcB* (LC777270), *ifcD1* (LC777271), and *ifcD2* (LC777272) are available in NCBI. The dataset supporting the results of this study is publicly available at the DDBJ (https://www.ddbj.nig.ac.jp) (PRJDB16018 and PRJDB16019). Additional data related to this paper will be made available from the corresponding author upon reasonable request.
