# Supplementary material for: An isoflavone catabolism gene cluster underlying interkingdom interactions in the soybean rhizosphere
Source: ISME Commun. 2024 Apr 9;4(1):ycae052. doi: 10.1093/ismeco/ycae052 (PMC11069340; doi:10.1093/ismeco/ycae052)
Supplement: Supplemental_Materials_ISME_v2_ycae052 [file supplemental_materials_isme_v2_ycae052.pdf]

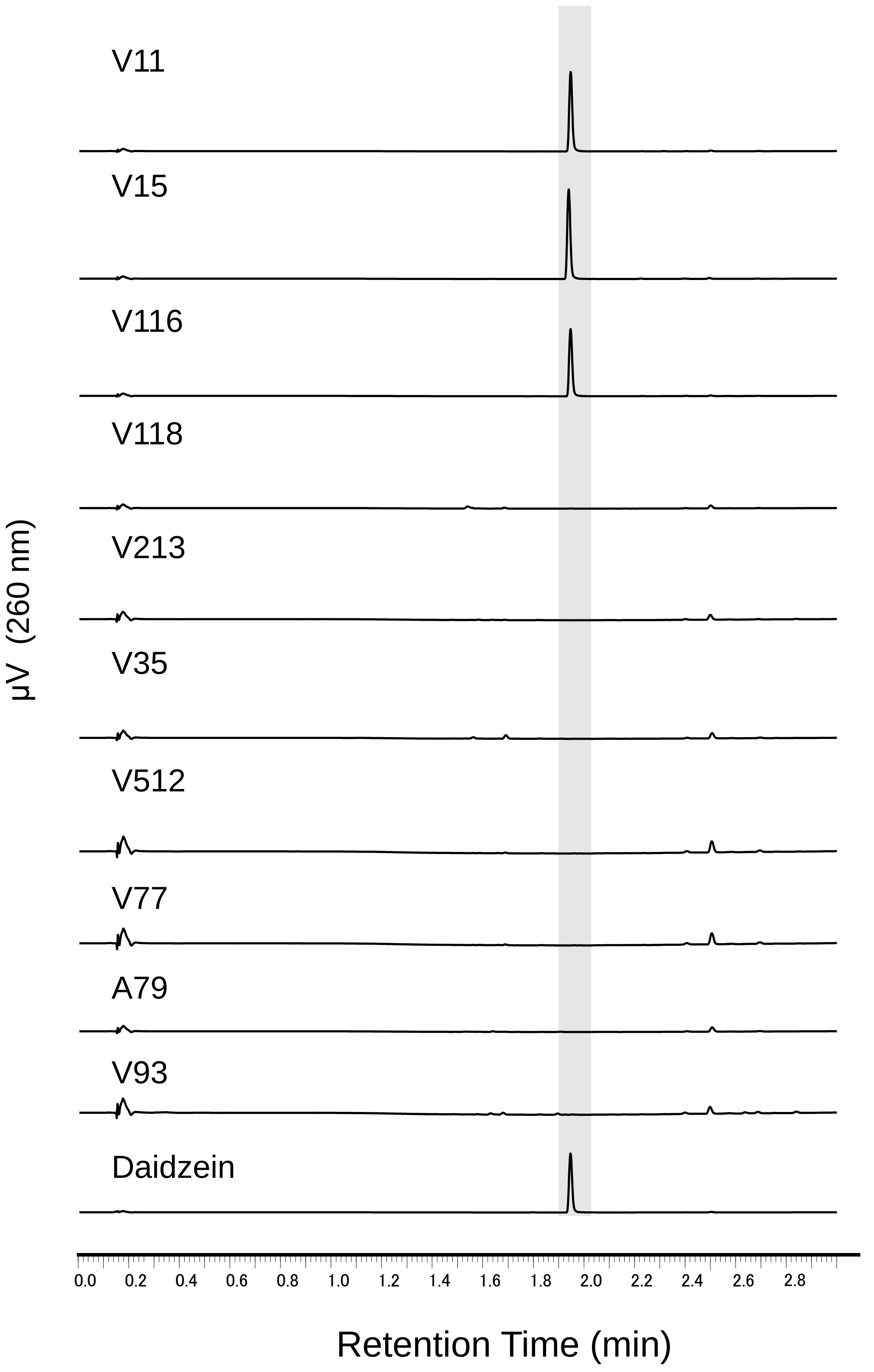

Fig. S1

Aoki et al.

(a)

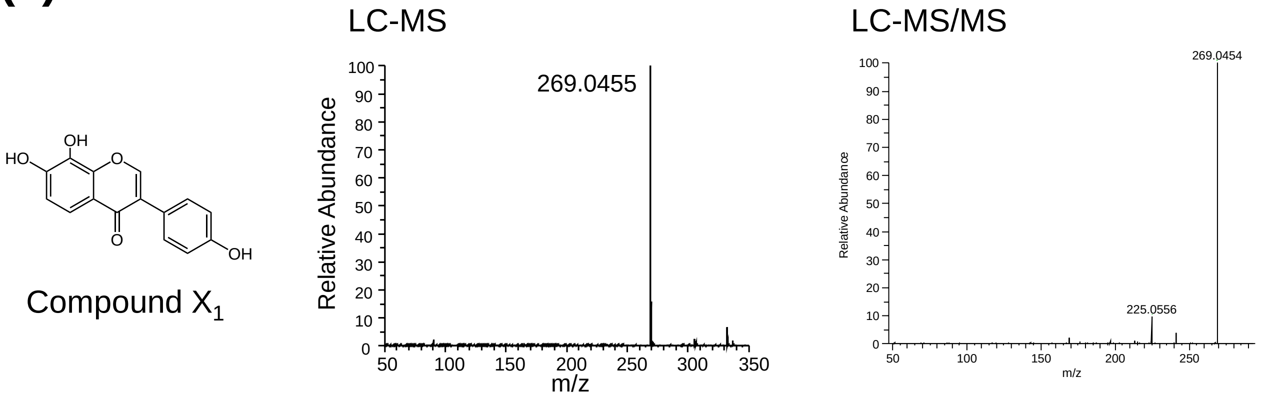

(b)

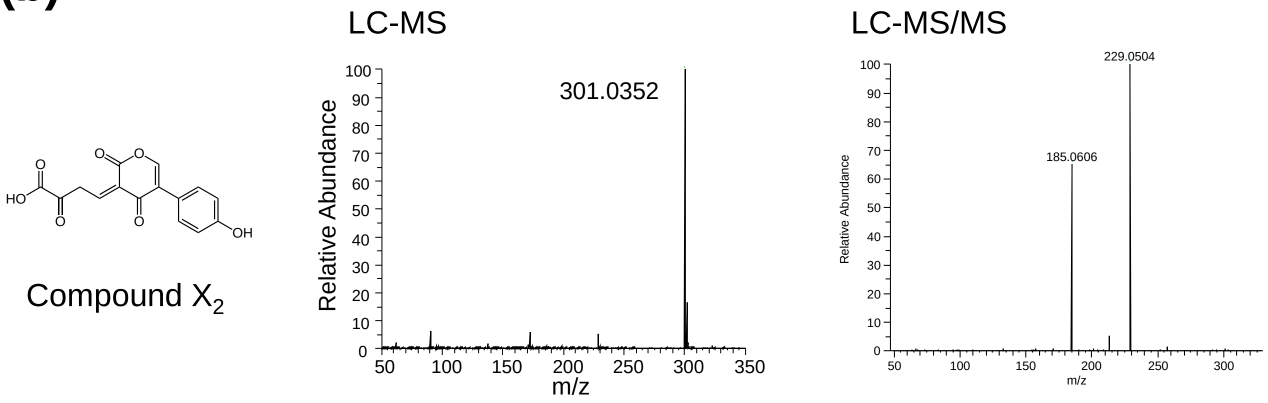

(c)

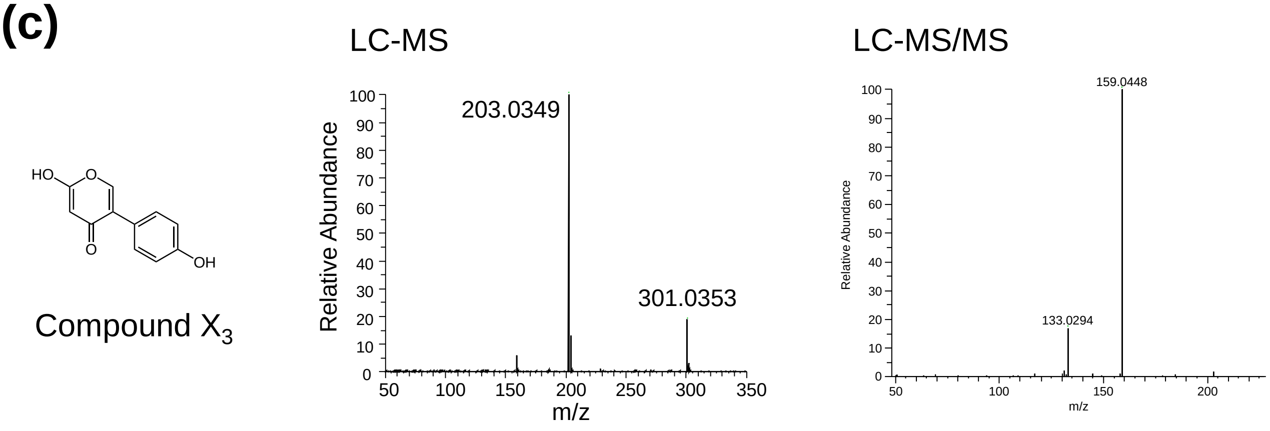

Fig. S2

Aoki et al.

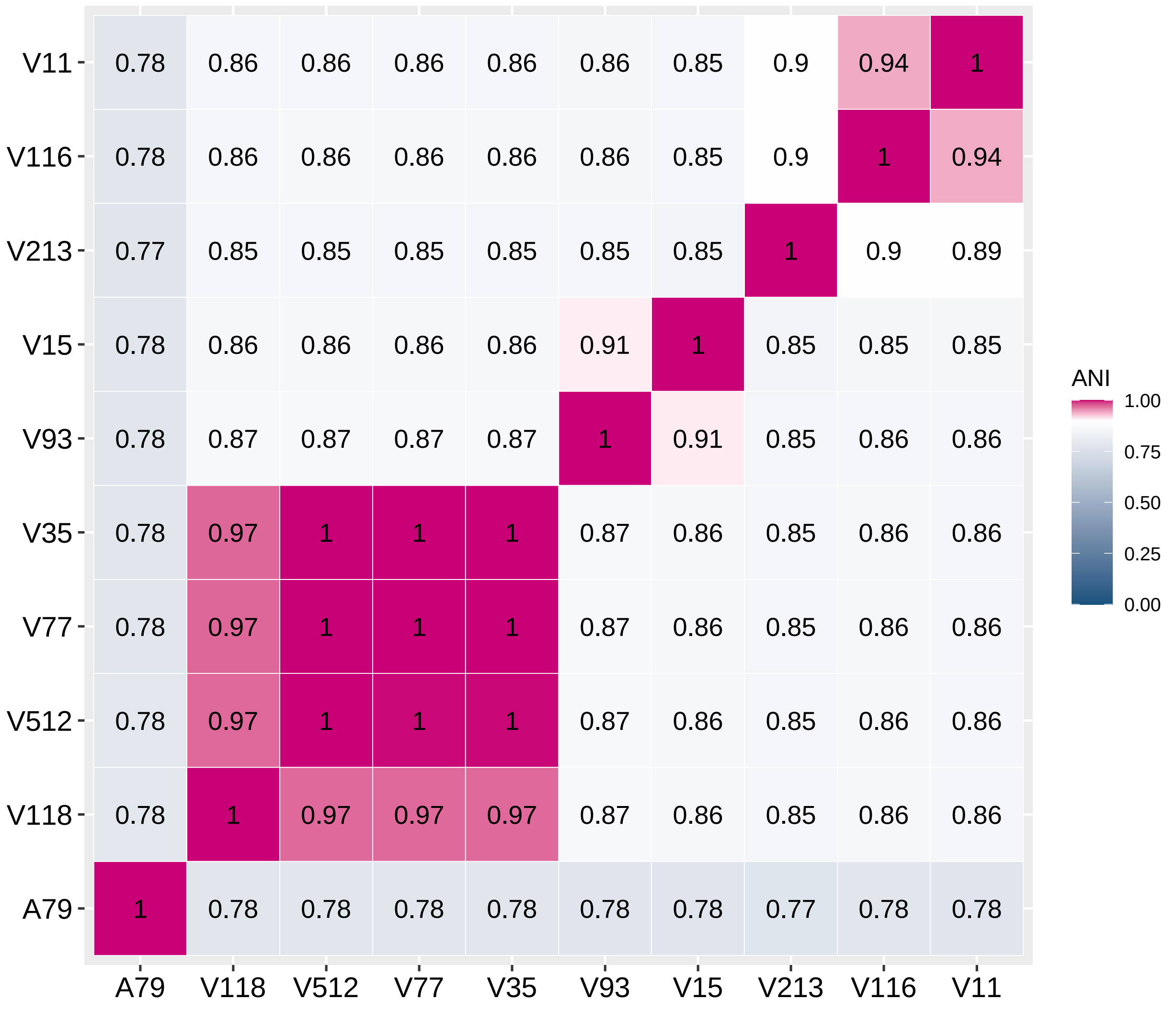

Fig. S3

Aoki et al.

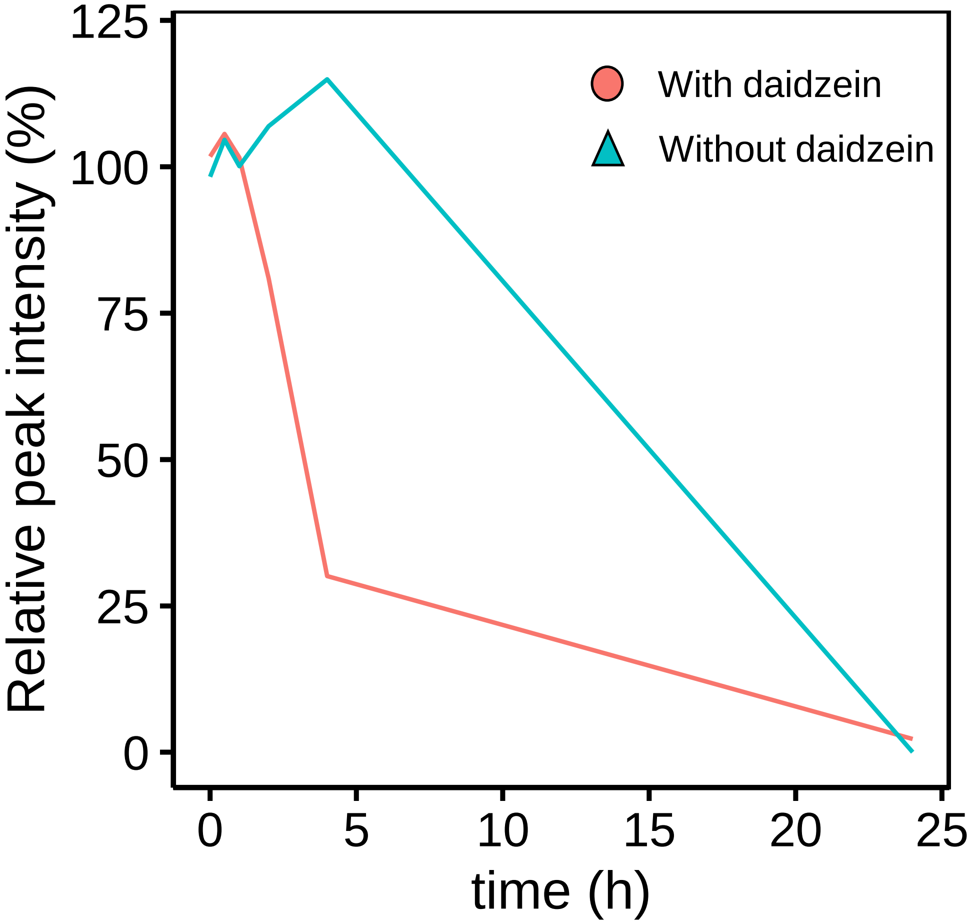

Fig. S4

Aoki et al.

(a)

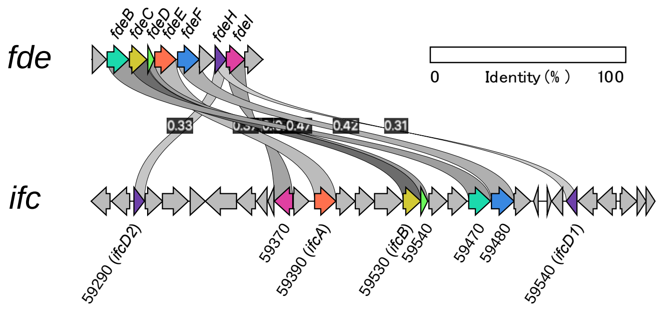

Gene	Annotation	Homologus gene (% identity)
fdeA	Protein involved in meta-pathway of phenol degradation	not hit
fdeB	alpha/beta hydrolase	59470 (48%)
fdeC	glyoxalase	59430 (66%)
fdeD	(2Fe-2S)-binding protein	59440 (55%)
fdeE	FAD-dependent 2-polyprenyl-6-methoxyphenol hydroxylase protein	59390 (36%)
fdeF	MFS transporter	59480 (45%)
fdeG	Cyclase	not hit
fdeH	cupin	59290 (37%)/ 59540 (35%)
fdeI	2-keto-4-pentenoate hydratase	59370 (45%)
fdeJ	UDP-glucose 4-epimerase	not hit

(b)

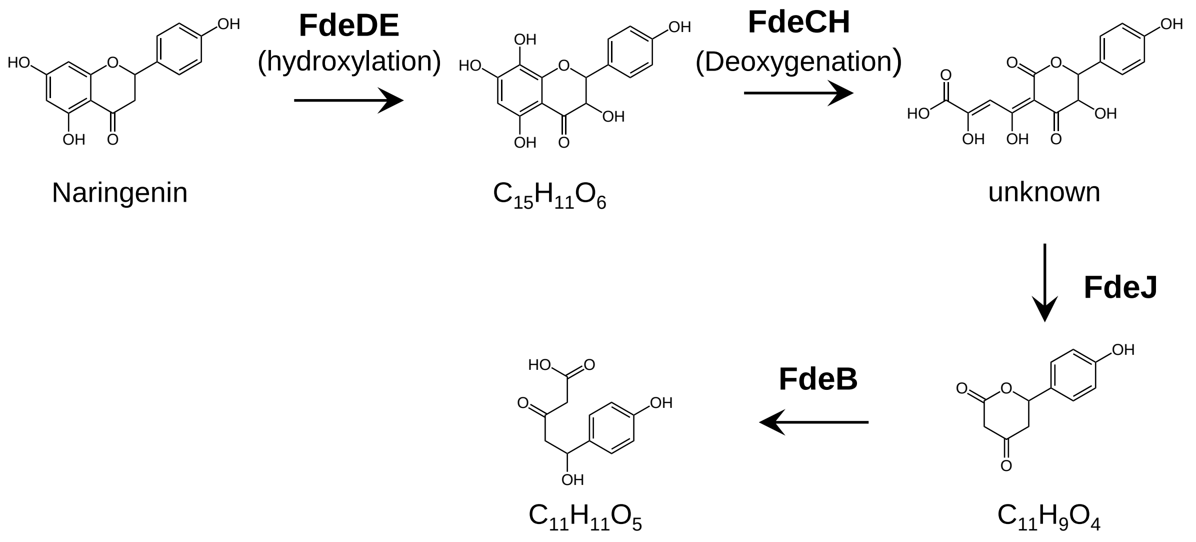

Fig. S5

Aoki et al.

TIC

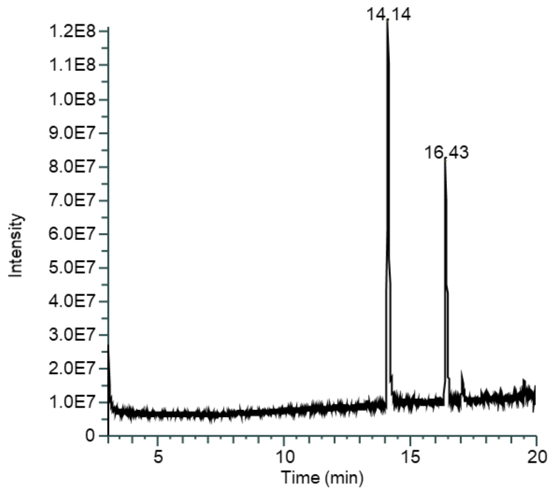

ms

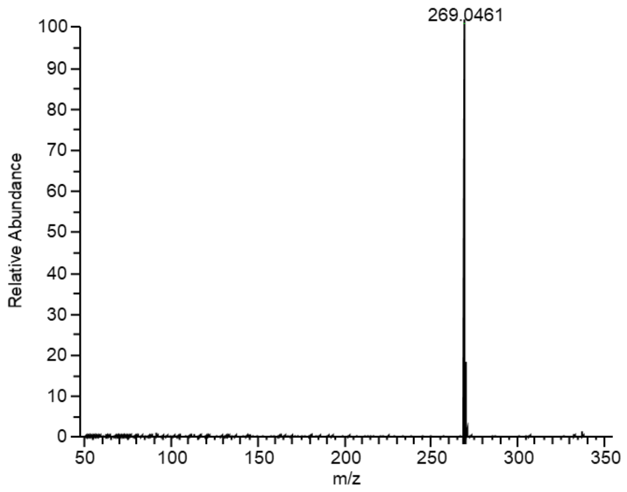

ms2

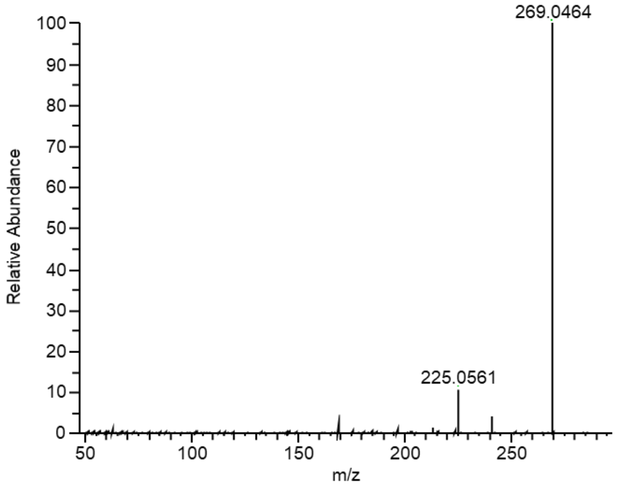

Fig. S6

Aoki et al.

TIC

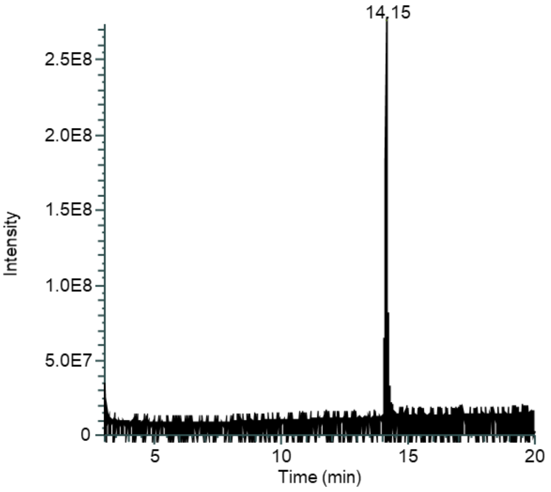

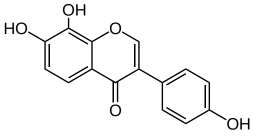

8-hydroxydaidzein

ms

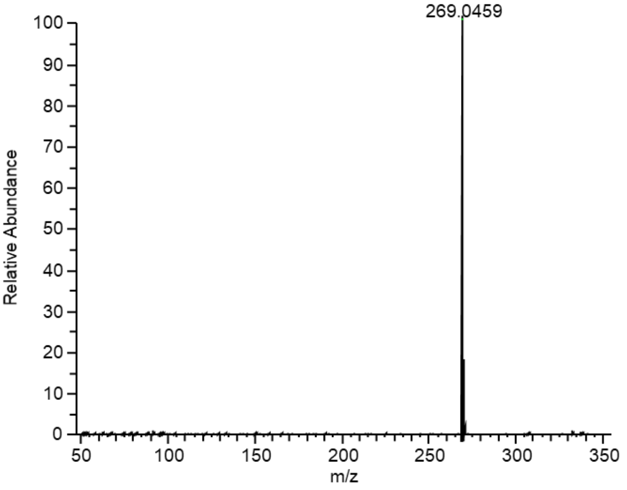

ms2

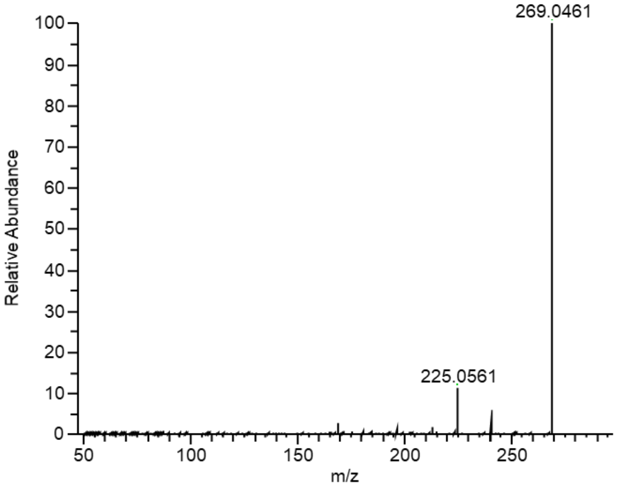

Fig. S7 Aoki et al.

TIC

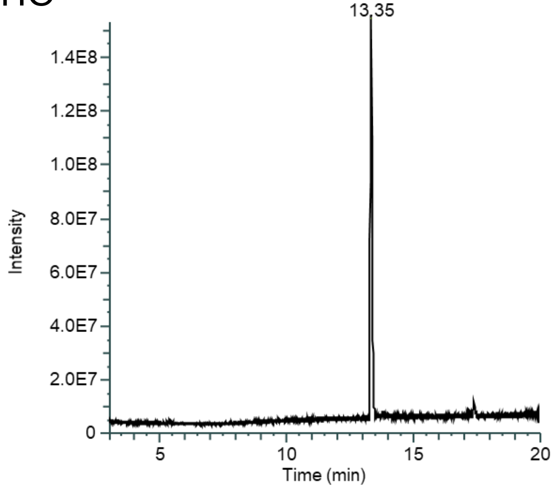

ms

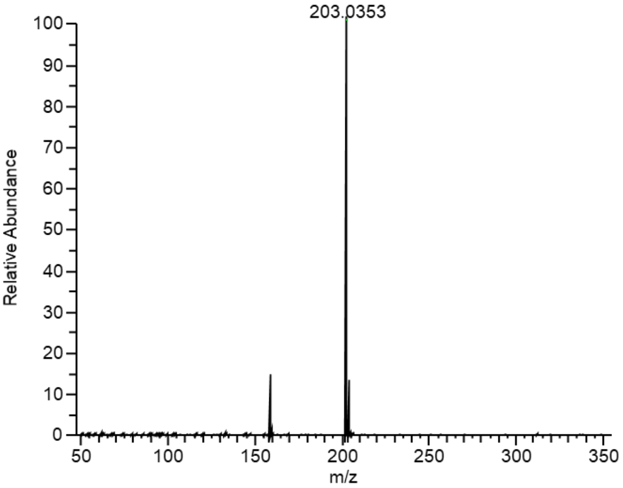

ms2

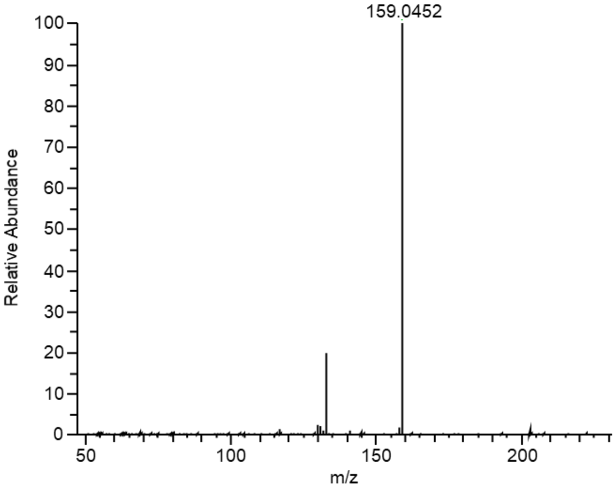

Fig. S8

Aoki et al.

(a)

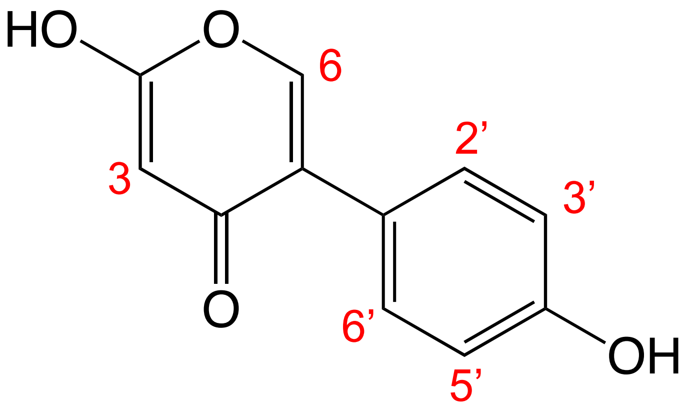

2-hydroxy-5-(4-hydroxyphenyl)-4*H*-pyran-4-one

(b)

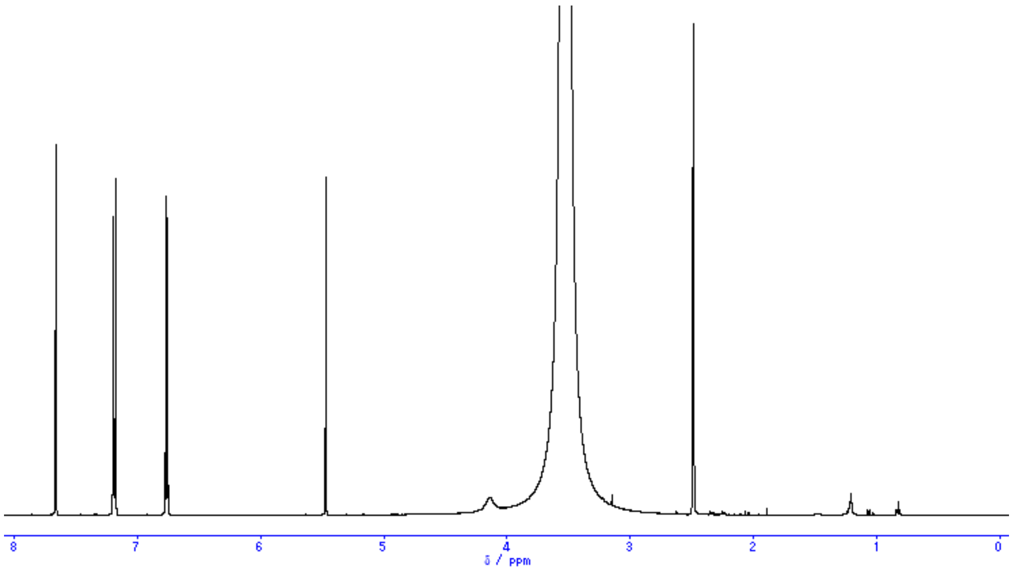

Fig. S9

Aoki et al.

(a) IfcA product

ms

ms2

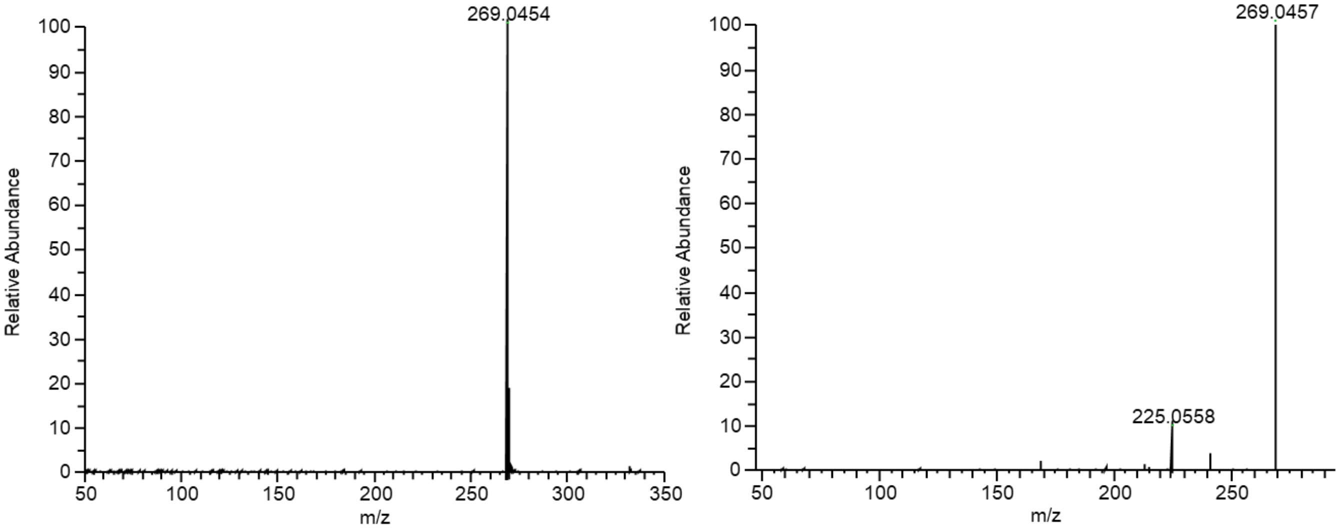

(b) 8-Hydroxydaidzein standard

ms

ms2

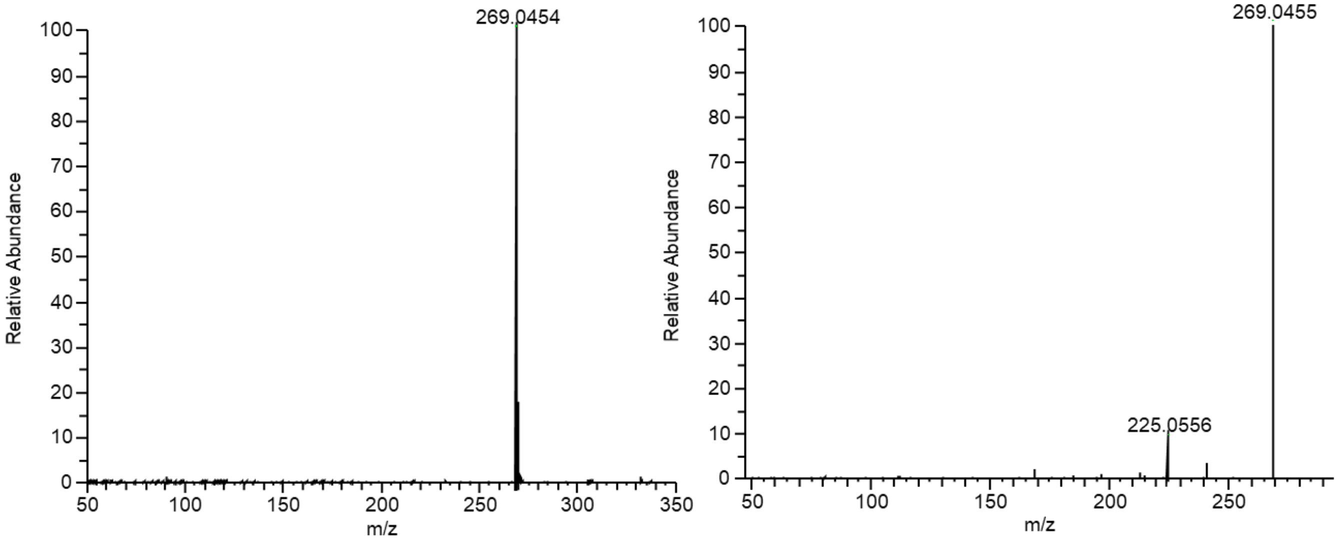

Fig. S10

Aoki et al.

IfcA + daidzein

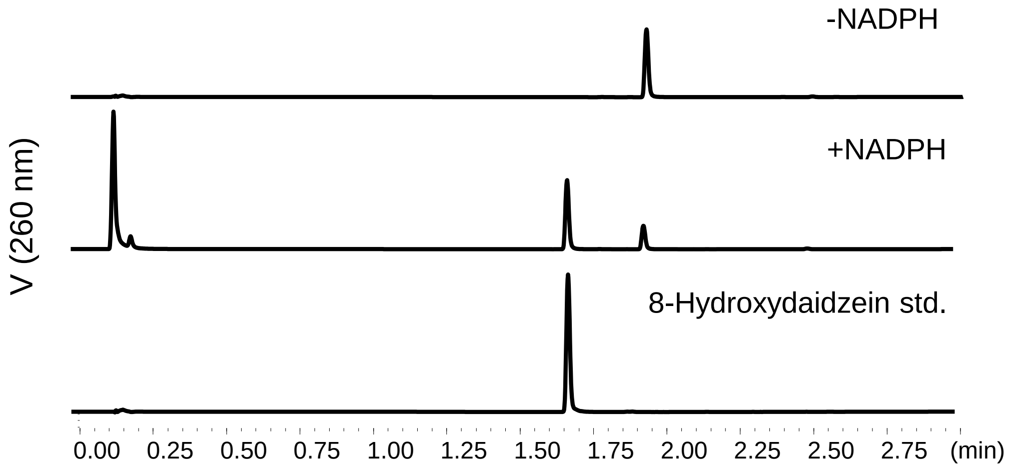

Fig. S11

Aoki et al.

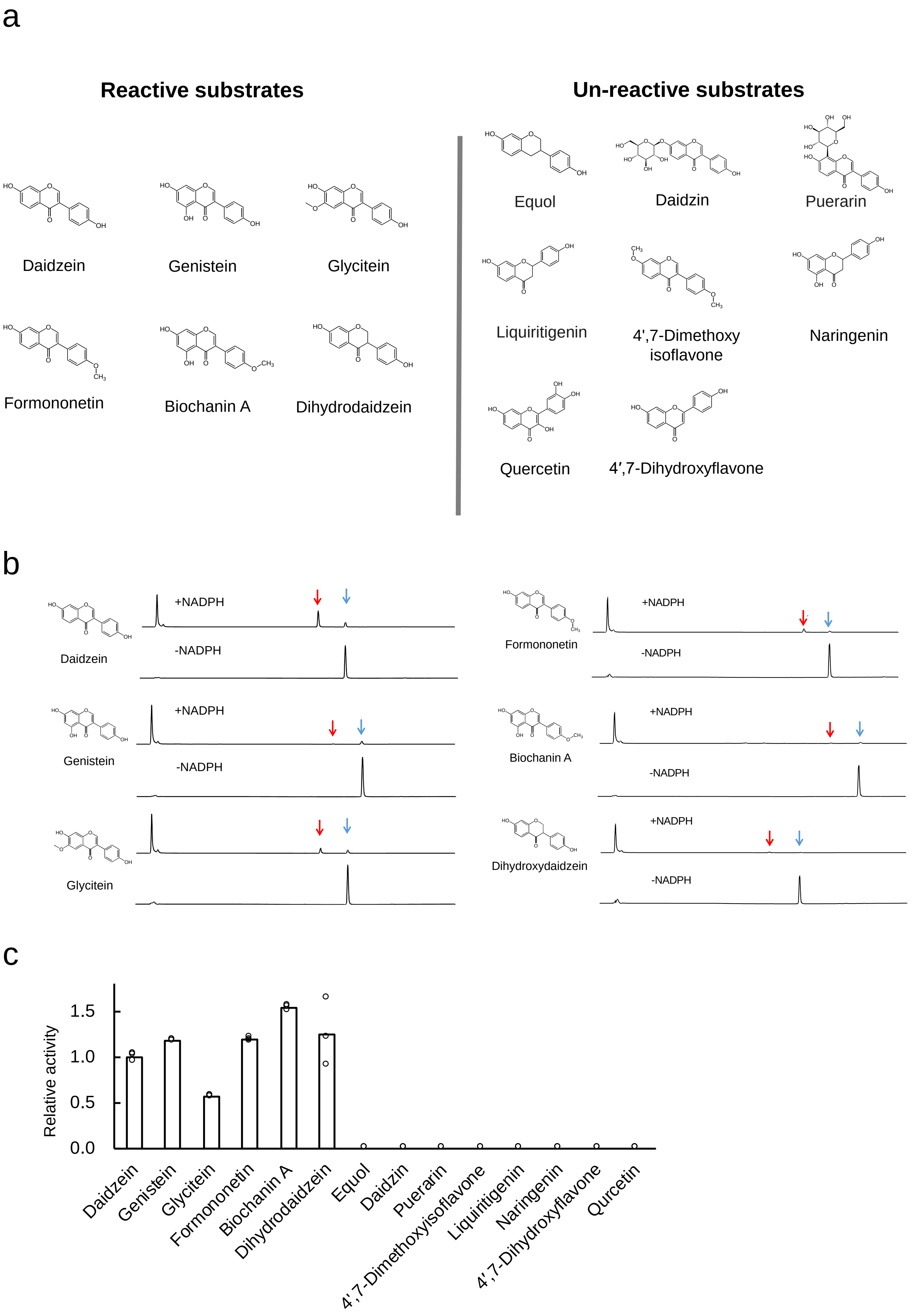

Fig. S12 Aoki et al.

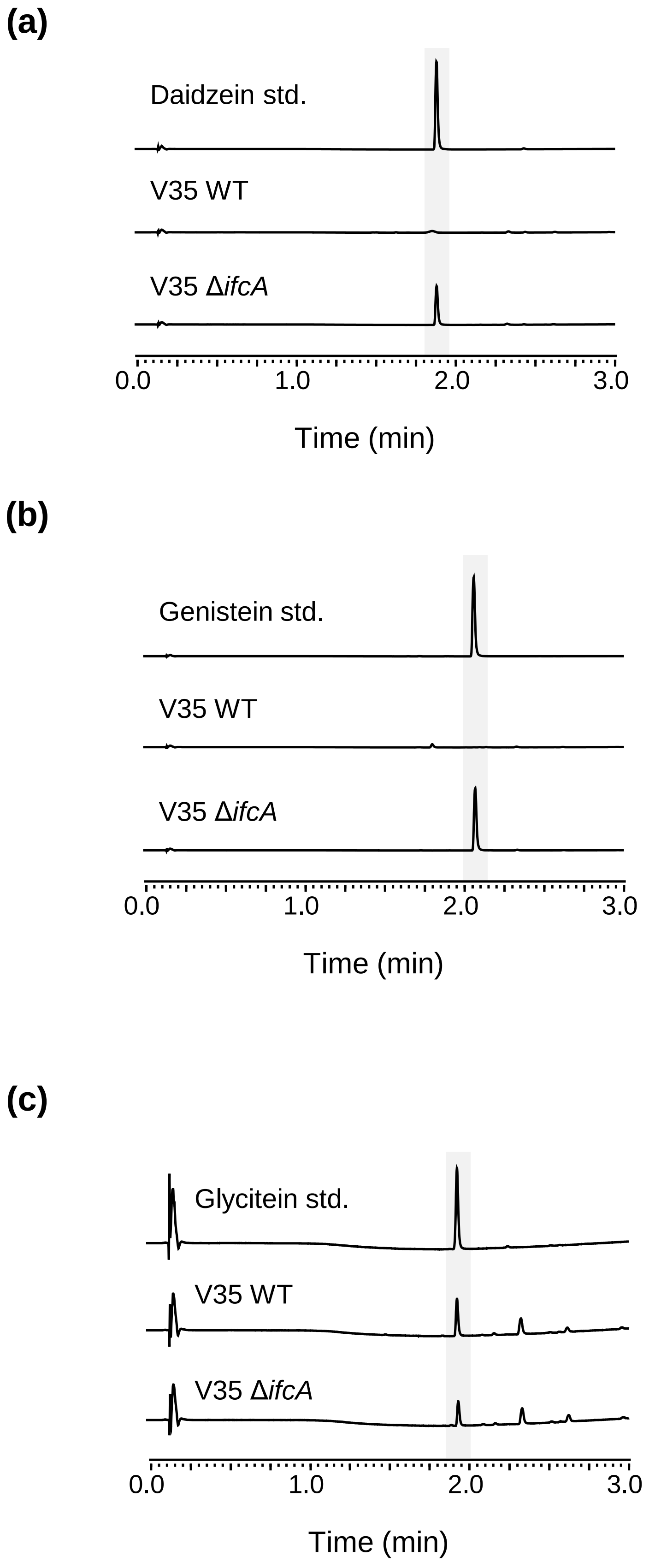

Fig. S13

Aoki et al.

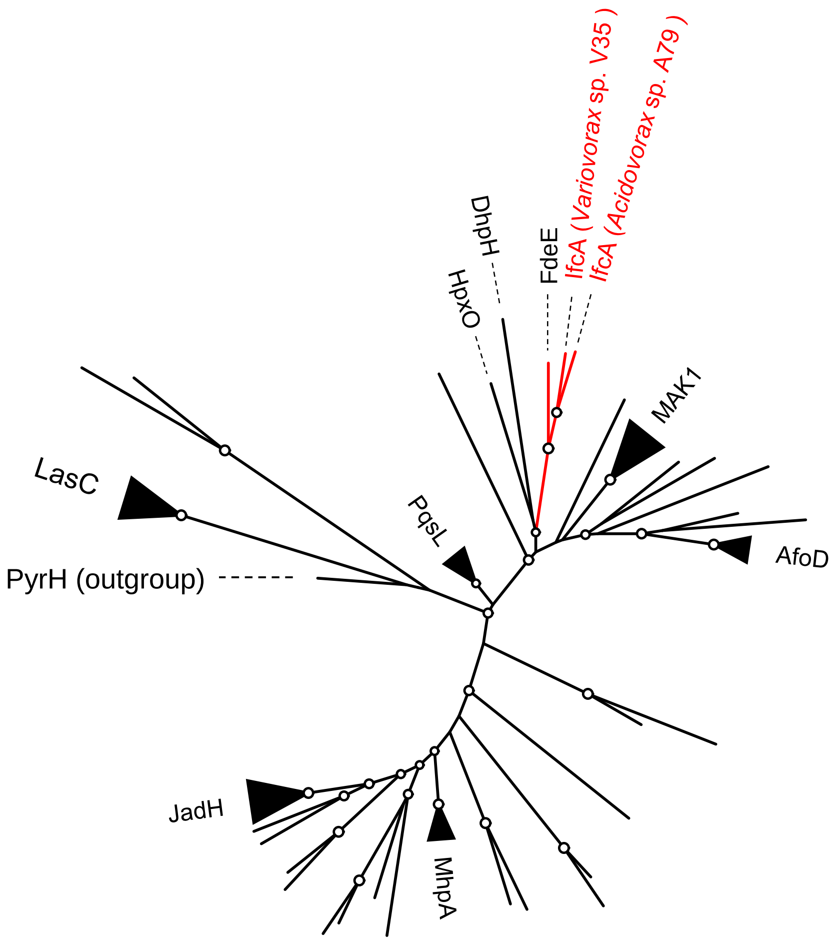

Fig. S14

Aoki et al.

(a) IfcB product

ms ms2

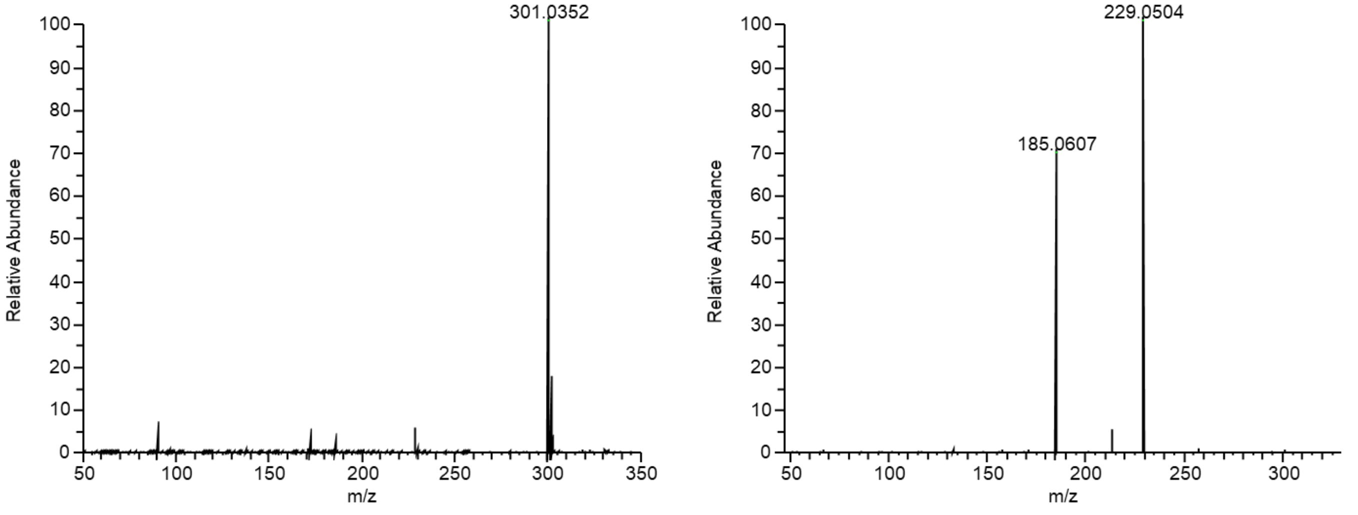

(b) Compound X_2

ms ms2

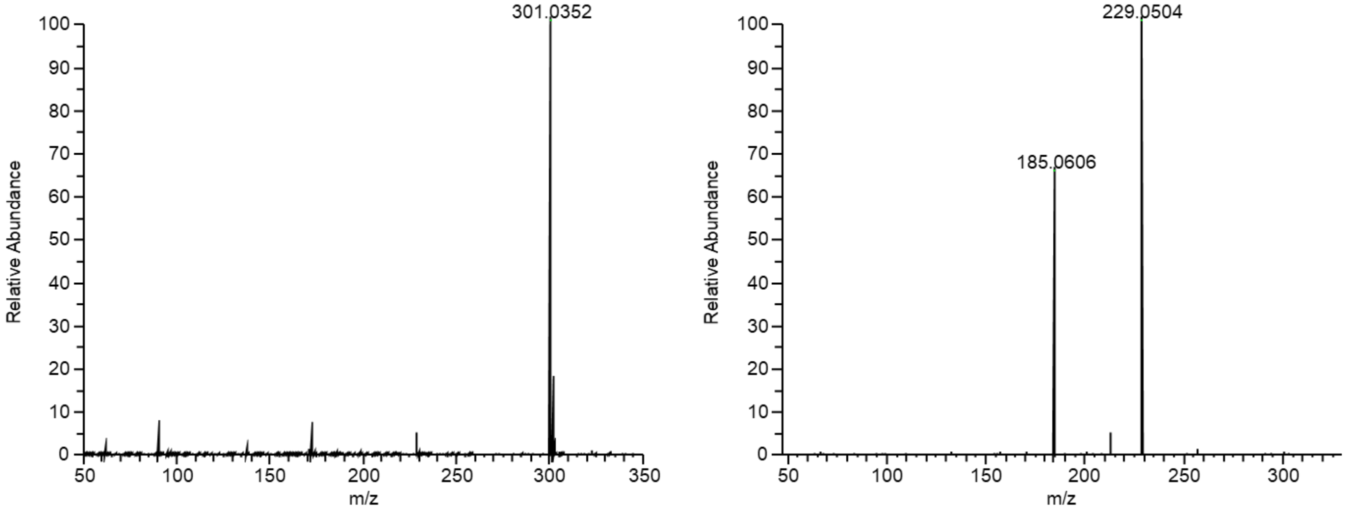

(a) IfcD1 product 1

ms

ms2

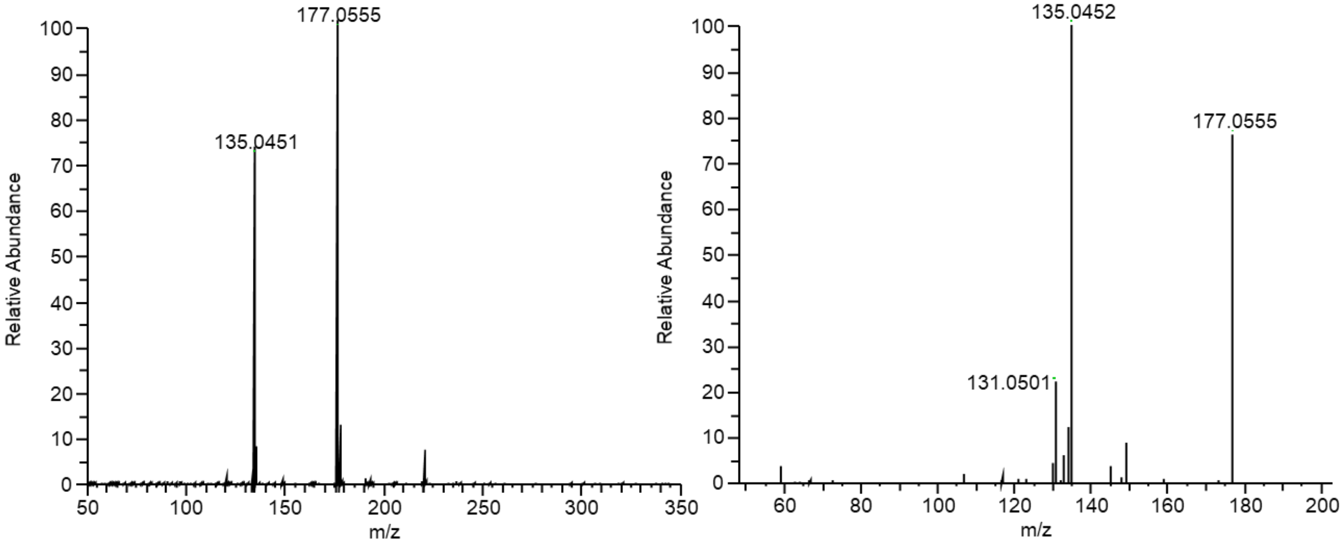

(b) IfcD1 product 2

ms

ms2

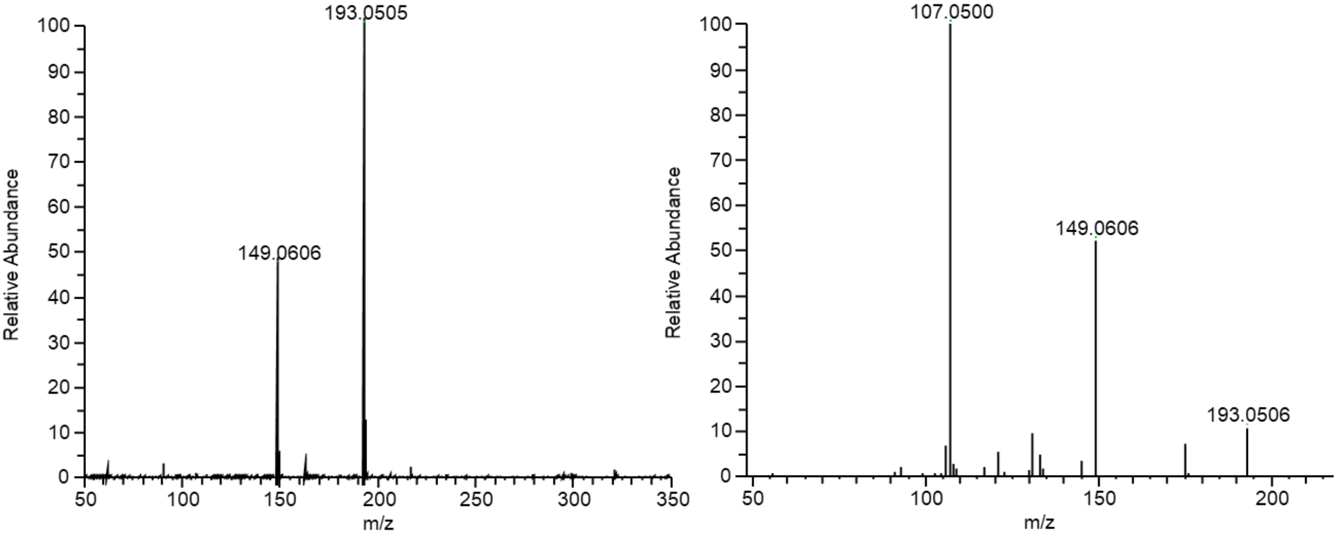

Fig. S16

Aoki et al.

(a) IfcD2 product 1

ms

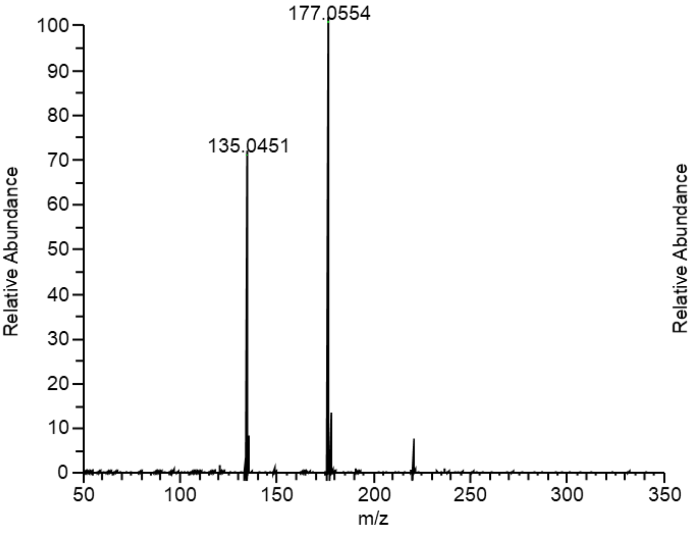

ms2

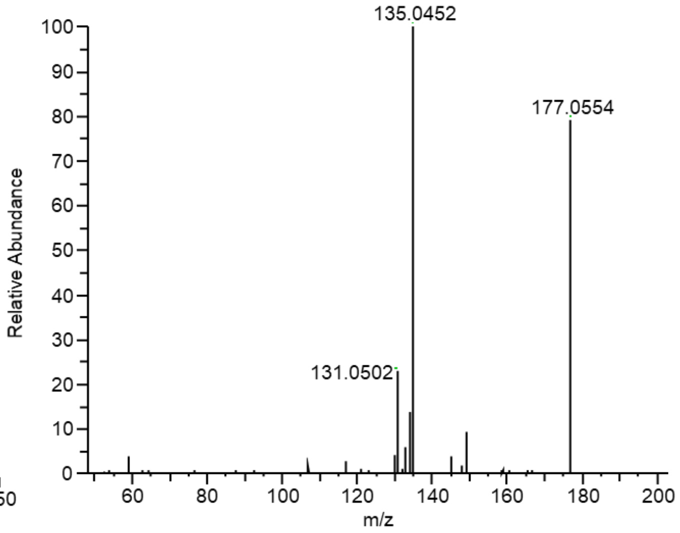

(b) IfcD2 product 2

ms

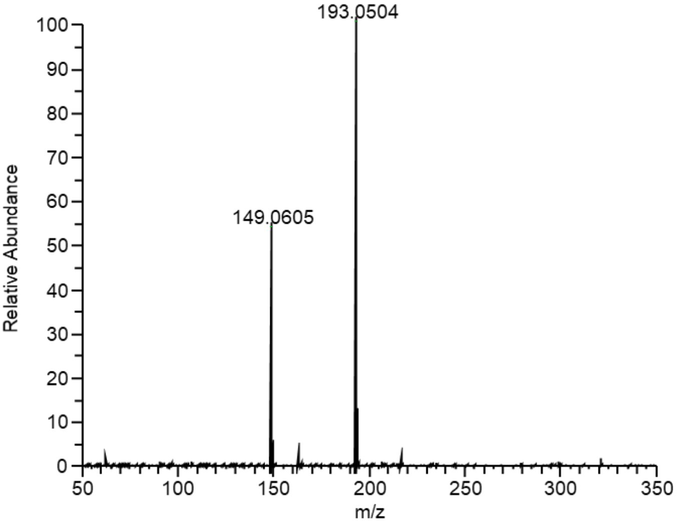

ms2

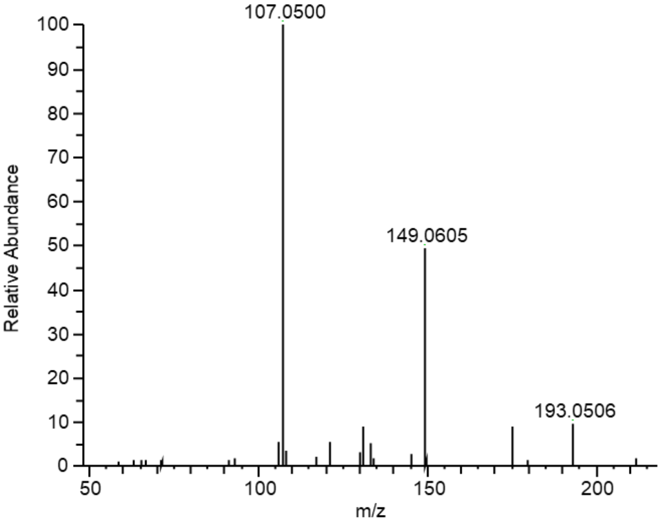

Fig. S17

Aoki et al.

(a) Compound X_3-d_2

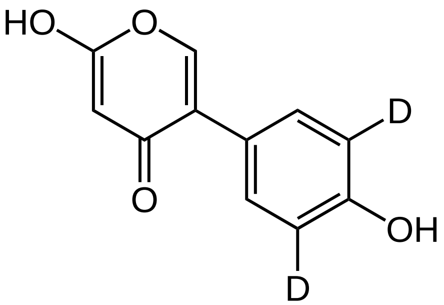

(b) IfcD1 product 1

ms

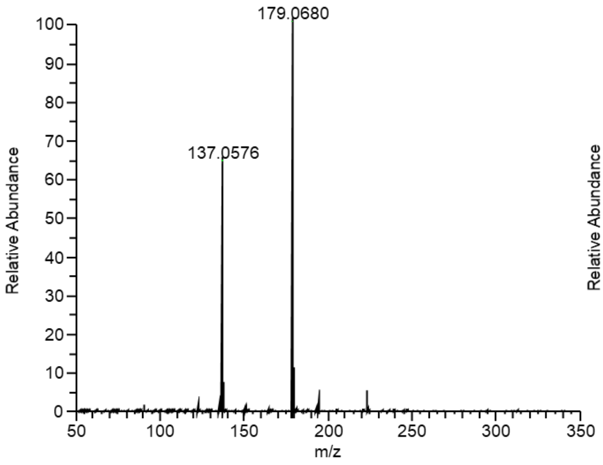

ms2

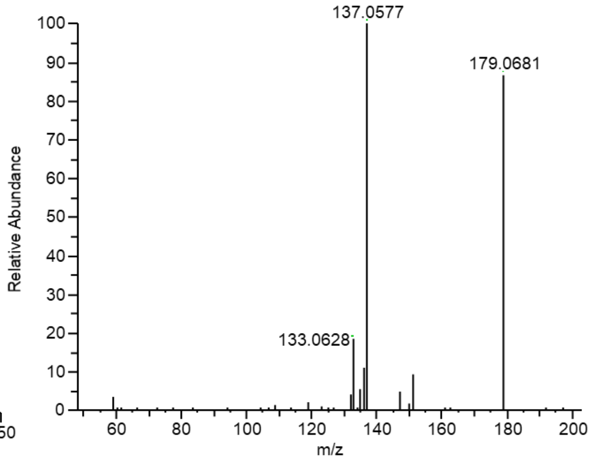

(C) IfcD1 product 2

ms

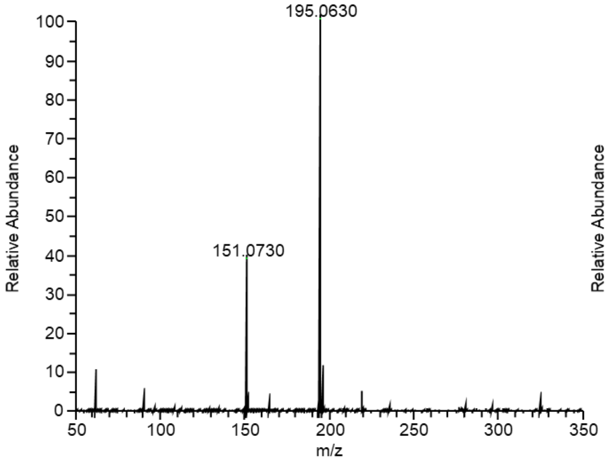

ms2

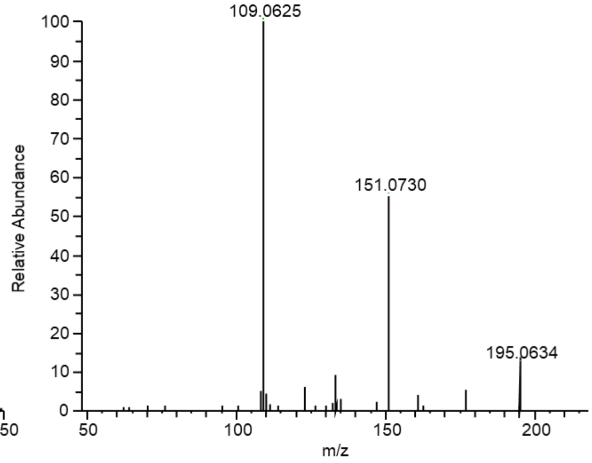

Fig. S18

Aoki et al.

(a) Compound X_3-d_4

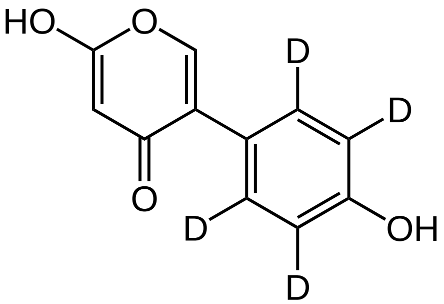

(b) IfcD1 product 1

ms

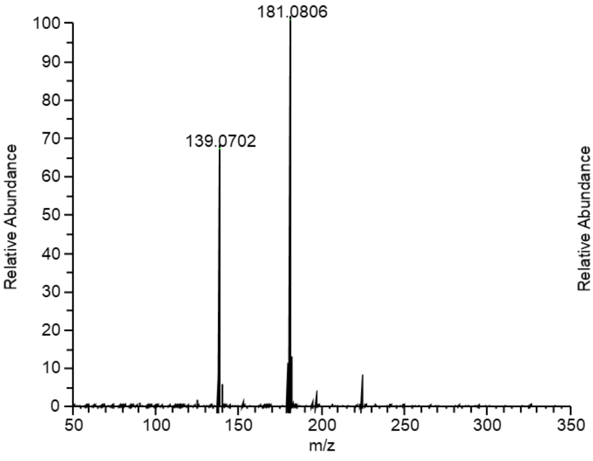

ms2

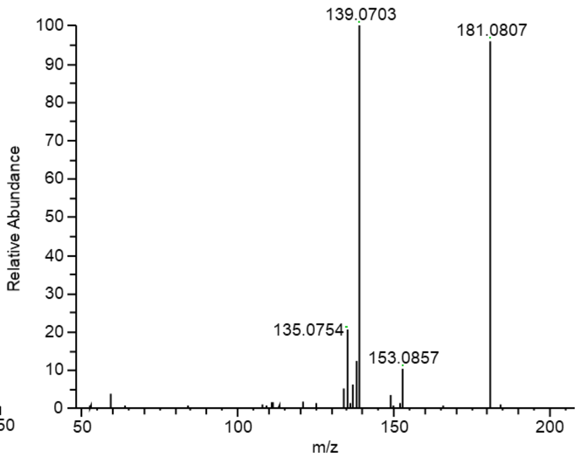

(C) IfcD1 product 2

ms

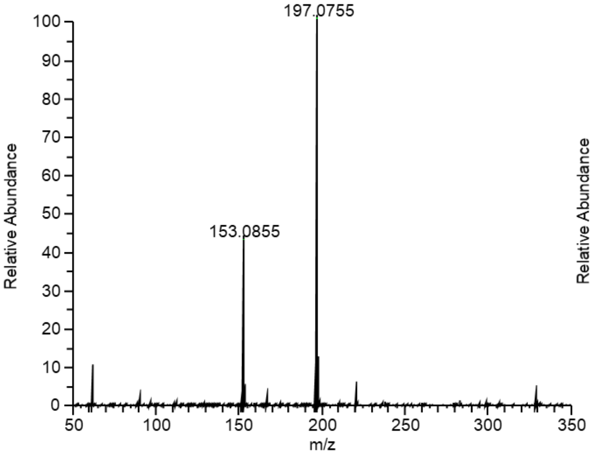

ms2

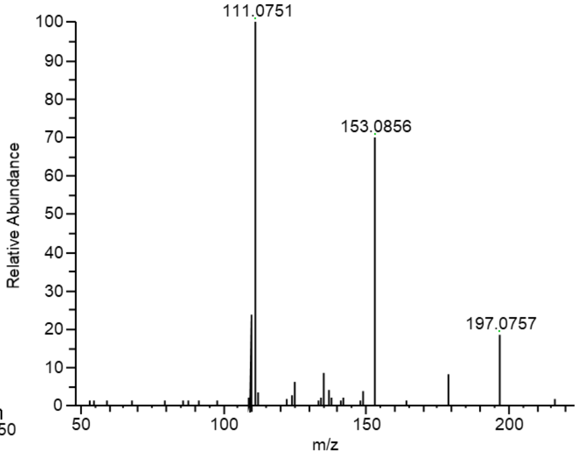

Fig. S19

Aoki ***et al.***,

(a) $C_{10}H_{10}O_3$

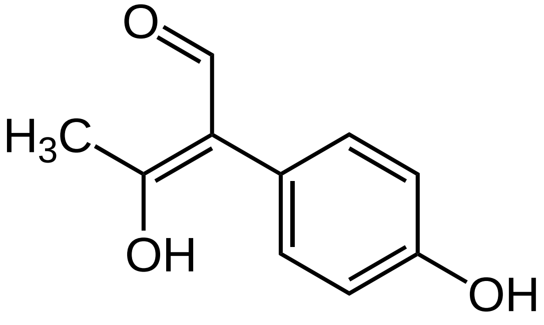

(b) $C_{10}H_{10}O_4$

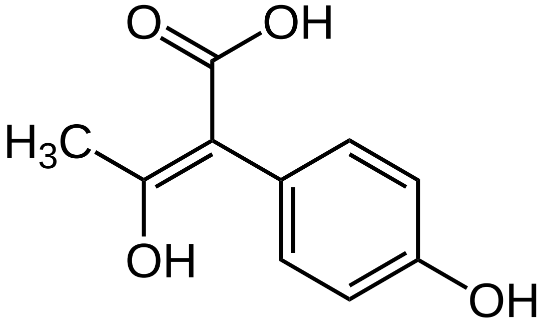

Fig. S20 Aoki et al.

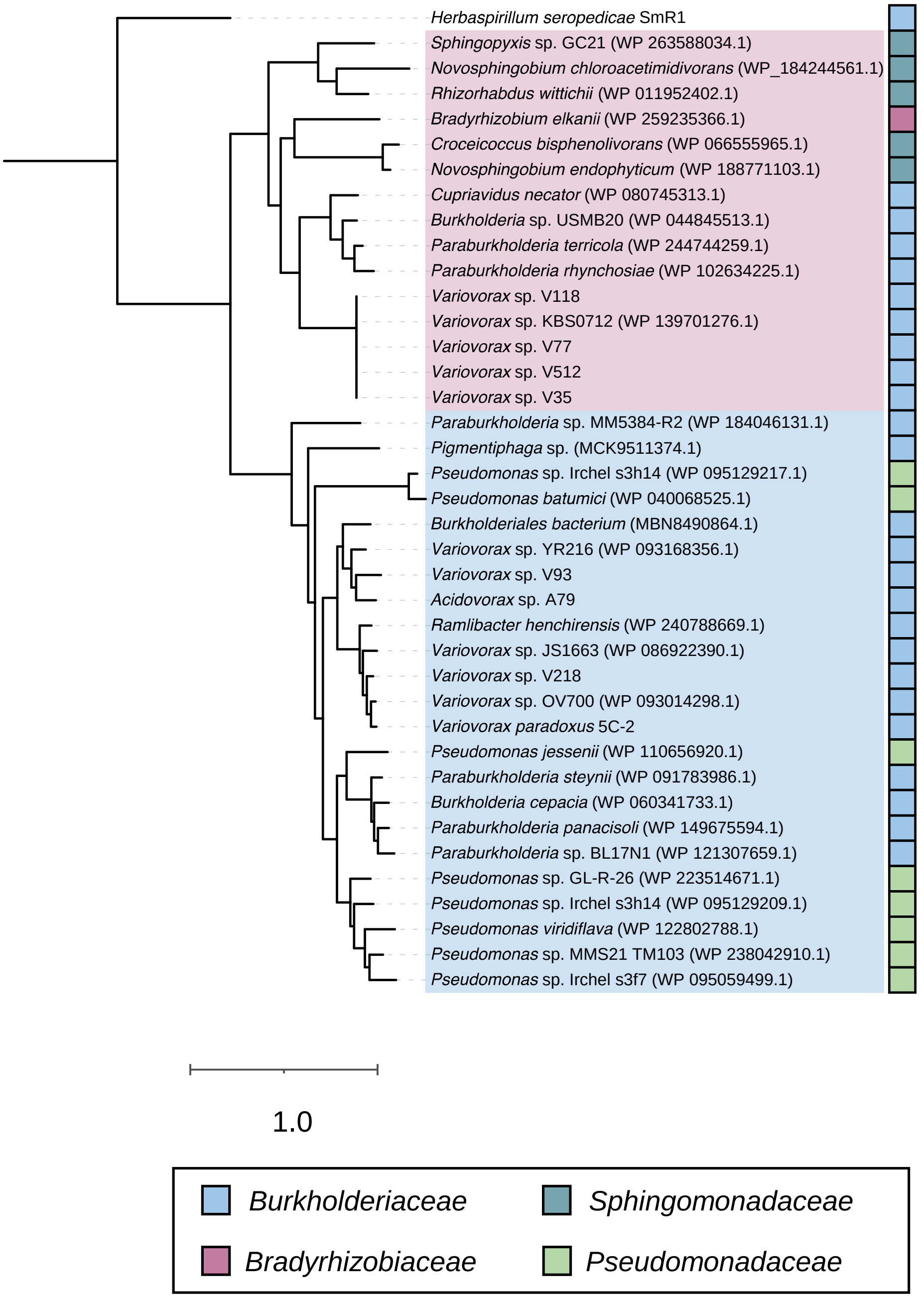

Fig. S21

Aoki et al.

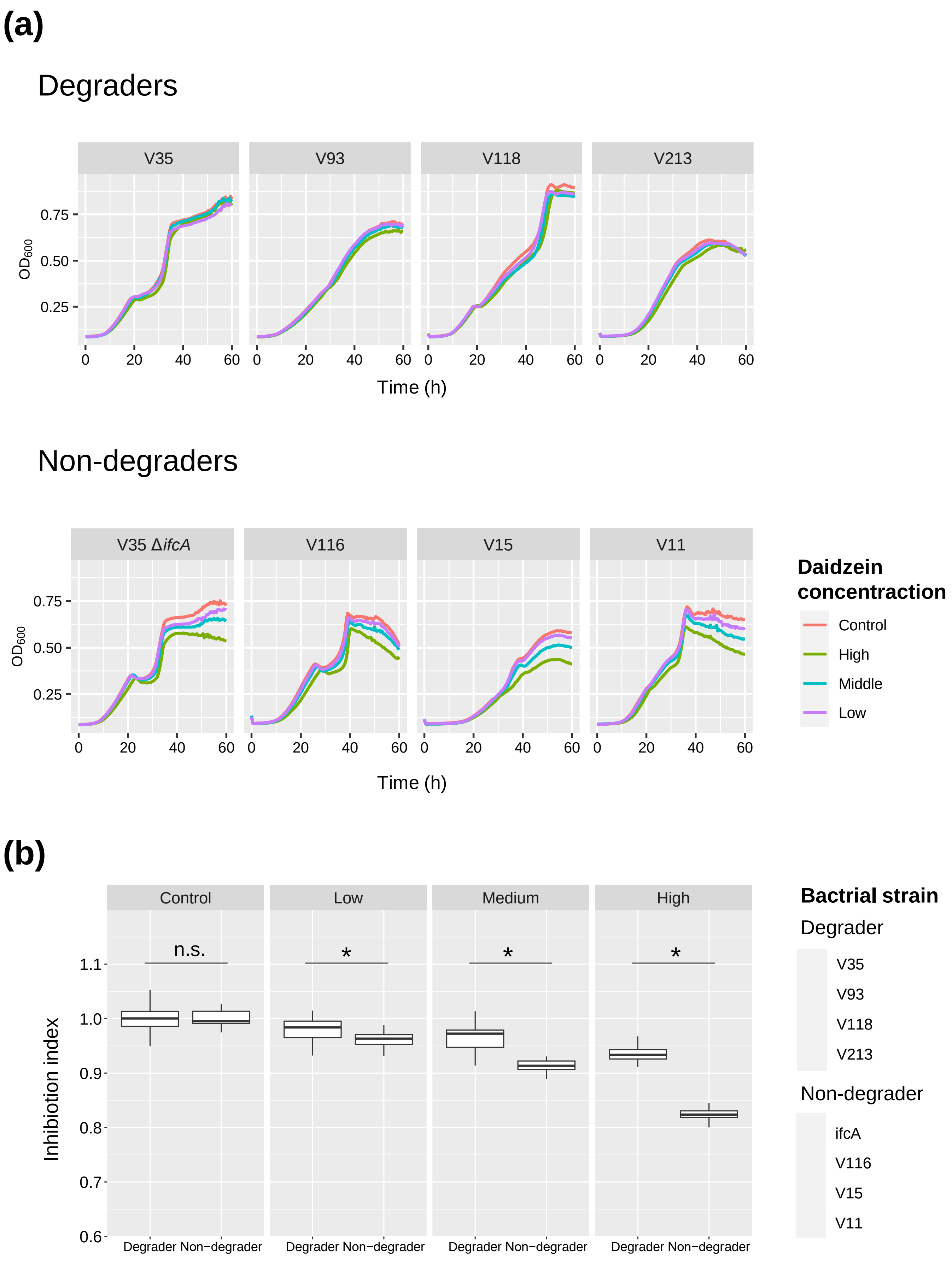

Fig. S22

Aoki et al.

(a)

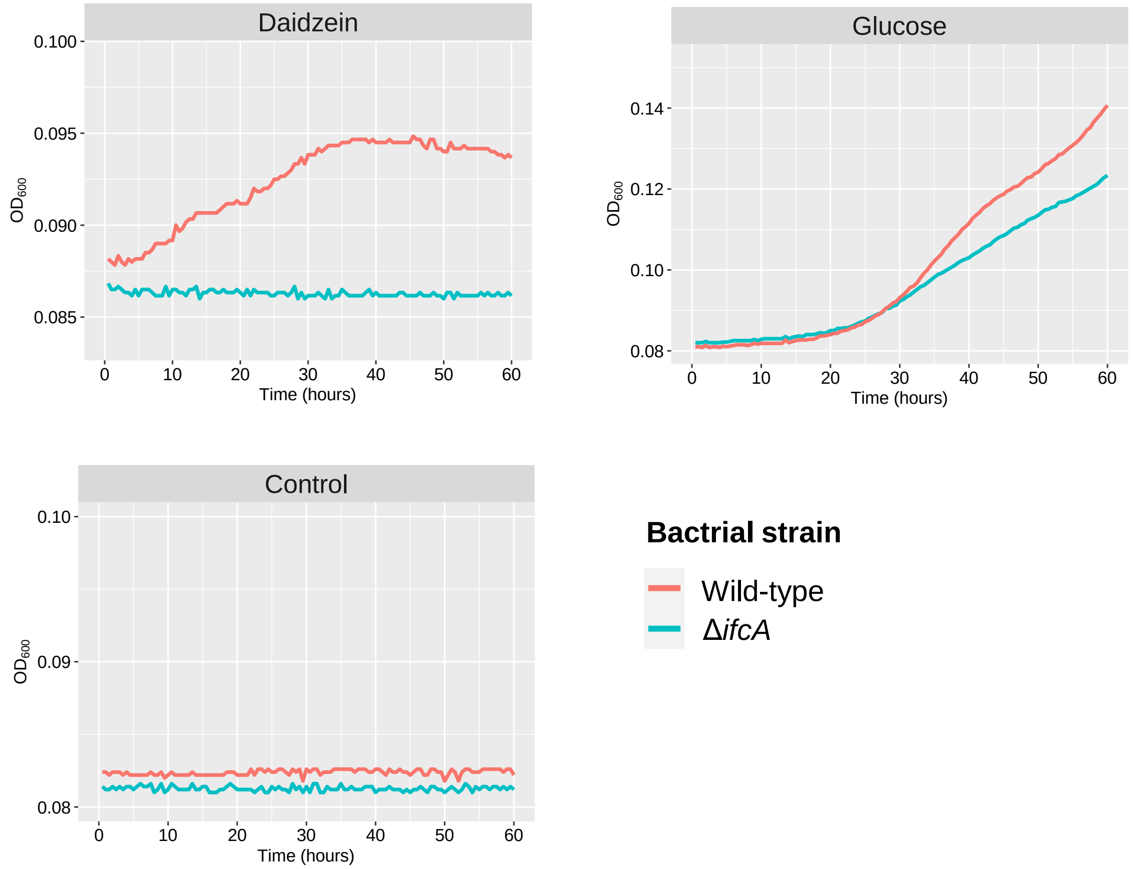

(b)

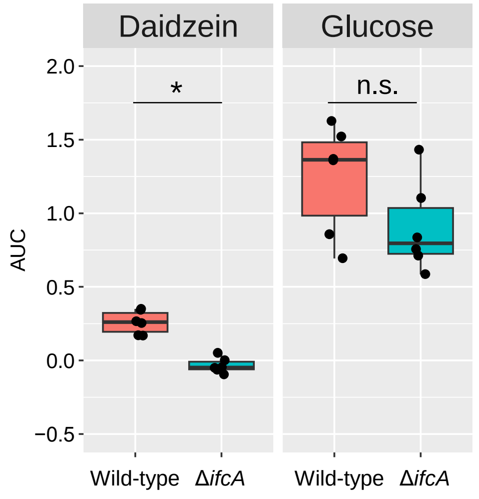

Fig. S23

Aoki et al.

Meta-cleavage of catechol pathway (M00569)

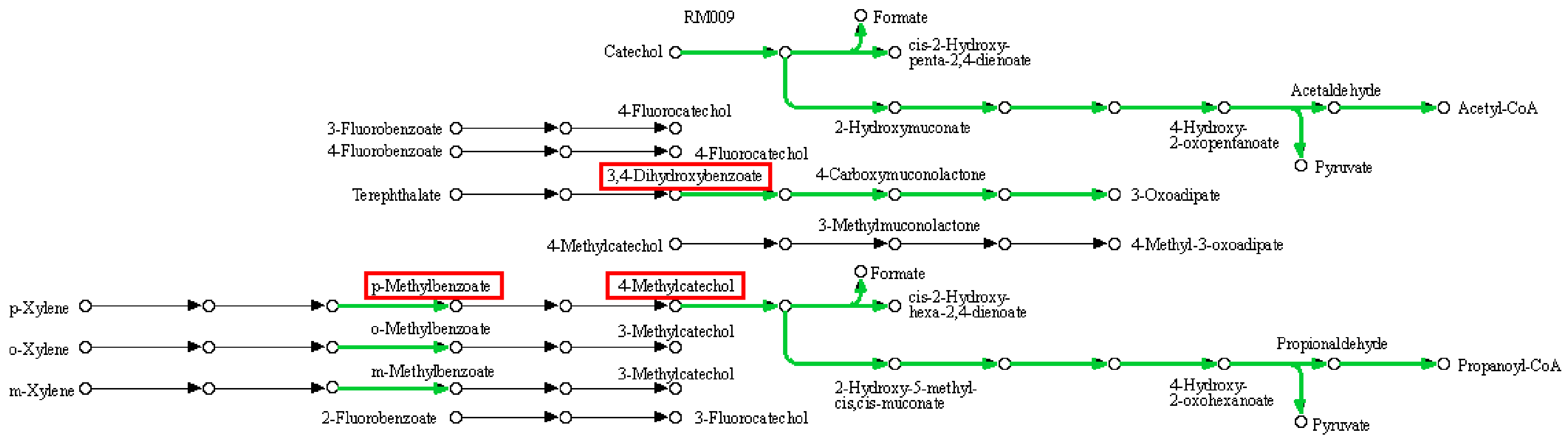

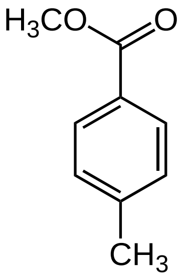

p-Methylbenzoate

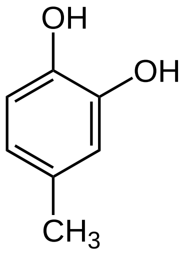

4-Methylcatechol

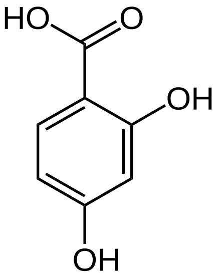

3,4-Dihydroxybenzoate

Aoki et al.

Fig. S24
